# Artesunate Impairs Growth in Cisplatin-Resistant Bladder Cancer Cells by Cell Cycle Arrest, Apoptosis and Autophagy Induction

**DOI:** 10.3390/cells9122643

**Published:** 2020-12-09

**Authors:** Fuguang Zhao, Olesya Vakhrusheva, Sascha D. Markowitsch, Kimberly S. Slade, Igor Tsaur, Jindrich Cinatl, Martin Michaelis, Thomas Efferth, Axel Haferkamp, Eva Juengel

**Affiliations:** 1 Department of Urology and Pediatric Urology, University Medical Center Mainz, Langenbeckstr. 1, 55131 Mainz, Germany; fzhao@uni-mainz.de (F.Z.); olesya.vakhrusheva@unimedizin-mainz.de (O.V.); Sascha.Markowitsch@unimedizin-mainz.de (S.D.M.); kimberlysue.slade@unimedizin-mainz.de (K.S.S.); igor.tsaur@unimedizin-mainz.de (I.T.); axel.haferkamp@unimedizin-mainz.de (A.H.); 2 Institute of Medical Virology, Goethe-University, 60596 Frankfurt am Main, Germany; cinatl@em.uni-frankfurt.de; 3 Industrial Biotechnology Centre, School of Biosciences, University of Kent, Canterbury CT2 7NJ, UK; M.Michaelis@kent.ac.uk; 4 Institute of Pharmaceutical and Biomedical Sciences, Johannes Gutenberg University Mainz, Staudinger Weg 5, 55128 Mainz, Germany; efferth@uni-mainz.de

**Keywords:** bladder cancer (BCa), artesunate (ART), cisplatin resistance, growth inhibition, apoptosis, autophagy

## Abstract

Cisplatin, which induces DNA damage, is standard chemotherapy for advanced bladder cancer (BCa). However, efficacy is limited due to resistance development. Since artesunate (ART), a derivative of artemisinin originating from Traditional Chinese Medicine, has been shown to exhibit anti-tumor activity, and to inhibit DNA damage repair, the impact of artesunate on cisplatin-resistant BCa was evaluated. Cisplatin-sensitive (parental) and cisplatin-resistant BCa cells, RT4, RT112, T24, and TCCSup, were treated with ART (1–100 µM). Cell growth, proliferation, and cell cycle phases were investigated, as were apoptosis, necrosis, ferroptosis, autophagy, metabolic activity, and protein expression. Exposure to ART induced a time- and dose-dependent significant inhibition of tumor cell growth and proliferation of parental and cisplatin-resistant BCa cells. This inhibition was accompanied by a G0/G1 phase arrest and modulation of cell cycle regulating proteins. ART induced apoptos is by enhancing DNA damage, especially in the resistant cells. ART did not induce ferroptosis, but led to a disturbance of mitochondrial respiration and ATP generation. This impairment correlated with autophagy accompanied by a decrease in LC3B-I and an increase in LC3B-II. Since ART significantly inhibits proliferative and metabolic aspects of cisplatin-sensitive and cisplatin-resistant BCa cells, it may hold potential in treating advanced and therapy-resistant BCa.

## 1. Introduction

Bladder cancer (BCa) is the second most common genitourinary malignancy [1] and the eleventh most common cancer worldwide [2]. In Western Europe almost 68,000 new cases of BCa and approximately 20,000 deaths were reported in 2018 [3]. BCa is particularly treacherous and treatment is cost-intensive, since even the relatively harmless non-muscle-invasive form, occurring in about 70% of cases at first presentation, often relapses after treatment and proceeds to invasive or systemic disease. Approximately a quarter of patients with BCa have muscle-invasive bladder cancer (MIBC) or metastatic disease [2]. Nearly half of MIBC cases, mainly patients with distant metastases, relapse after radical cystectomy [2]. For physically fit patients with advanced or metastatic bladder cancer, but with good renal function (~50%), chemotherapy based on the DNA damaging agent cisplatin is standard first-line treatment. However, the efficacy of cisplatin-based therapy is limited due to the development of therapy resistance. Cisplatin resistance often occurs due to enhanced DNA damage repair pathway activity, e.g., nucleotide excision repair, mismatch repair, and the Fanconi Anemia pathway [4]. Moreover, alterations in drug transporters contribute to inhibition of drug influx and/or increased drug efflux, in turn leading to diminished drug content within the tumor cells [4,5]. Adduct formation with glutathione (GSH), methionine, metallothioneins, and other cytoplasmic nucleophiles inactivate cisplatin, maintaining a lower drug concentration within the nucleus [4,6]. Furthermore, metabolic reprogramming is pivotal in cisplatin resistance [7]. Thus, these mechanisms re-activate cell cycling, tumor growth, and cell survival, and prevent induction of apoptosis, promoting cisplatin resistance [4,7]. Cisplatin-ineligible, but PD-L1-positive patients with advanced/metastatic bladder cancer can be treated with a carboplatin-based chemotherapy or an immune checkpoint inhibitor. Erdafitinib, a fibroblast growth factor receptor inhibitor (FGFR), has also been approved by the U.S. Food and Drug Administration for patients with metastatic bladder cancer exhibiting actionable FGFR alteration [8]. However, even these additional first-line treatments rarely result in complete or durable remission, due to acquired therapy resistance. Thus, a great number of patients benefit only temporarily from chemo- and/or immunotherapy [9]. Therefore, novel therapy options are urgently needed.

Artesunate (ART), a semi-synthetic water-soluble derivative of artemisinin, extracted from *Artemisia annua* (sweet wormwood) in Traditional Chinese Medicine, was originally clinically developed for the treatment of malaria. In addition, ART also exhibited profound anti-tumor activity, induced DNA damage and inhibited DNA damage repair. ART rendered cervical cancer cells [10] and esophageal cancer cells [11] susceptible to radiotherapy by inhibiting DNA damage repair. ART inhibited the RAD51 and ATM/ATR damage response, affecting DNA damage repair in ovarian cancer [12,13]. Moreover, ART sensitized neuroblastoma [14], ovarian [12], and head and neck cancer to cisplatin in vitro [15,16] by inducing oxidative stress DNA double-strand breaks [12,13], apoptosis [14] and ferroptosis [15]. As an anti-malaria agent, ART induced reactive oxygen species (ROS) due to the high iron content in the *Plasmodium* parasites, thereby destroying the malaria pathogen [17,18] by ferroptosis, an iron- and ROS-dependent controlled cell death. Tumor cells also contain more iron than normal cells [19,20], most probably due to highly expressed transferrin receptors [19,20,21,22,23,24], which might, at least partially, explain why ART specifically affects some tumor cells [25,26,27,28]. In doxorubicin-resistant T-cell leukemia and cisplatin-resistant neuroblastoma, ART provoked the generation of reactive oxygen species (ROS), contributing to apoptosis induction [14,29]. Besides inducing ferroptosis [15,16,30] and mitochondrial apoptosis [31,32,33,34], ART also triggered autophagy [32,35,36]. Thus, ART exhibited multifarious anti-tumor activity. However, ART’s impact on bladder cancer cells has not been investigated in detail and its effect on therapy-resistant bladder cancer is still unknown. This study was designed to determine the efficacy of ART on a panel of therapy-sensitive (=parental) and cisplatin-resistant bladder cancer cells in vitro and evaluate its mode of action.

## 2. Materials and Methods

### 2.1. Cell Cultures

The cell lines RT4, RT112, T24, and TCCSup were obtained from DSMZ. The cisplatin-resistant sublines were derived from the RCCL collection (https://research.kent.ac.uk/industrial-biotechnology-centre/the-resistant-cancer-cell-line-rccl-collection/) [37]. RT4 (grade 1), RT112 (grade 2/3), T24 (grade 3), and TCCSup (grade 4) represent transitional cell carcinomas. BCa cells RT4 were grown and sub-cultured in Iscove Basal medium (Biochrom GmbH, Berlin, Germany), RT112, T24, and TCCSup in RPMI-1640 medium (Gibco, Thermo Fisher Scientific, Darmstadt, Germany). The media were supplemented with 10% fetal calf serum (FCS) (Gibco, Thermo Fisher Scientific, Darmstadt, Germany), 1% glutamax (Gibco, Thermo Fisher Scientific, Darmstadt, Germany), and 1% Anti-Anti (Gibco, Thermo Fisher Scientific, Darmstadt, Germany). Next, 20 mM HEPES-buffer (Sigma-Aldrich, Darmstadt, Germany) was added to the RPMI-1640 medium. Tumor cells were cultivated in a humidified, 5% CO_2_ incubator.

### 2.2. Resistance Induction and Drug Treatment

Resistance sublines were established by continuous exposure to stepwise increasing drug concentrations as previously described [38]. The cisplatin-resistant tumor cells were exposed to 1 μg/mL cisplatin (Selleckchem, Munich, Germany) three times a week. Therapy-sensitive tumor cells served as controls. The cisplatin-resistant BCa cells were designated as RT4res, RT112res, T24res, and TCCSupres, the parental controls as RT4par, RT112par, T24par, and TCCSuppar. The half-maximal inhibitory concentration (IC50) of cisplatin in the tumor cells was investigated to verify drug resistance. ART (Sigma-Aldrich, Darmstadt, Germany) was applied for 24, 48, or 72 h at a concentration of 1–100 μM. Controls (parental and cisplatin-resistant) remained ART-untreated. To evaluate toxic effects of ART, cell viability was determined by trypan blue (Sigma-Aldrich, Darmstadt, Germany). Ferrostatin-1 (Sigma-Aldrich, Darmstadt, Germany), the ferroptosis inhibitor, was used at a concentration of 20 μM. Hydroxycloroquine (Sigma-Aldrich, Darmstadt, Germany), an inhibitor of autophagosome and lysosome fusion, was applied at a concentration of 20 µM for the last 3 h of the experiment.

### 2.3. Tumor Cell Growth

Cell growth was assessed using a 3-(4,5-dimethylthiazol-2-yl)-2,5-diphenyltetrazolium bromide (MTT) dye. BCa cells (50 μL, 1 × 10^5^ cells/mL) were seeded onto 96-well-plates. After 24, 48, and 72 h, 10 μL MTT (0.5 mg/mL) was added for 4 h. Cells were then lysed in 100 µL solubilization buffer containing 10% SDS in 0.01 M HCl. The 96-well-plates were then incubated overnight at 37 °C, 5% CO_2_. Absorbance at 570 nm was determined for each well using a microplate enzyme-linked immunosorbent assay (ELISA) reader (Tecan, Spark 10 M, Crailsheim, Germany). After subtracting background absorbance and offsetting with a standard curve, results were calculated as mean cell number and expressed as a percentage. To illustrate the kinetics of dose-response, 24 h data were set to 100%. Each experiment was done in triplicate.

### 2.4. Proliferation

Cell proliferation was measured using a BrdU (5-bromo-2′-deoxyuridine) cell proliferation enzyme-linked immunosorbent assay (ELISA) kit (Calbiochem/Merck Biosciences, Darmstadt, Germany). Tumor cells (50 μL, 1 × 10^5^ cells/mL), seeded onto 96-well-plates, were incubated with 20 μL BrdU-labeling solution per well for 24 h and then fixed and stained using anti-BrdU mAb according to the manufacturer’s protocol. Absorbance was measured at 450 nm using a microplate ELISA reader (Tecan, Spark 10 M, Crailsheim, Germany). Values were presented as percentage compared to untreated controls set to 100%.

### 2.5. Cell Cycle Phase Distribution

For cell cycle analysis, cell cultures were grown to sub-confluency. A total of 1 × 10^6^ cells was stained with propidium iodide (Invitrogen, Thermo Fisher Scientific, Darmstadt, Germany) and then subjected to flow cytometry (Fortessa X20, BD Biosciences, Heidelberg, Germany). From each sample, 10,000 events were collected. Data acquisition was carried out using DIVA software (BD Biosciences, Heidelberg, Germany) and cell cycle distribution was analyzed by ModFit LT 5.0 software (Verity Software House, Topsham, ME, USA). The number of cells in the G0/G1, S, or G2/M phases was expressed as the percentage of the total cell number.

### 2.6. Apoptosis and Necrosis

To investigate apoptotic and necrotic events, the FITC-Annexin V Apoptosis Detection kit (BD Biosciences, Heidelberg, Germany) was used to quantify binding of Annexin V/propidium iodide (PI). After washing tumor cells twice with PBS, 1 × 10^5^ cells were suspended in 500 μL of binding buffer and incubated with 5 μL Annexin V-FITC and (or) 5 μL PI in the dark for 15 min. Staining was measured by flow cytometer (Fortessa X20, BD Biosciences, Heidelberg, Germany). From each sample, 10,000 events were collected. The percentage of apoptotic and necrotic cells in each quadrant was calculated using DIVA software (BD Biosciences, Heidelberg, Germany). Further analysis was done by FlowJo software (BD Biosciences, Heidelberg, Germany).

### 2.7. Evaluation of Mitochondrial Respiration and Anaerobic Glycolytic Activity

Mitochondrial respiration (OCR = oxygen consumption rate) and anaerobic glycolytic activity (EACR = extracellular acidification rate) was assessed in real time by the Seahorse XFp Extracellular Flux Analyzer using the Seahorse XF Cell Mito Stress Test Kit (both: Agilent Technologies, Waldbronn, Germany). The EACR indicating anaerobic glycolytic activity was used to determine compensatory glycolysis. OCR was obtained by multiple parameters, including basal respiration, ATP production-coupled respiration, maximal and reserve capacities, and non-mitochondrial respiration. Cells stained with CellTracker Green CMFDA (Thermo Fisher Scientific, Darmstadt, Germany) were plated at a density of 2 × 10^4^ cells/well and media replaced with XF Assay media the following day 1 h prior to the assay and incubated without CO_2_. Five measurements of OCR and ECAR were taken at baseline and after each injection of the following mitochondrial modulators: oligomycin (1.5 µM, Inhibitor of ATP synthase), carbonylcyanide 4-(trifluoromethoxy)- phenylhydrazone (FCCP) (1 µM, proton gradient uncoupler) and rotenone/actinomycin A (0.5 µM, inhibitors of complex I/Complex III). Data were normalized by using Wave 2.6.1 desktop software to the mean fluorescent intensity of cells in the area of measurement in each well. Data pertaining to the OCR were normalized to total basal respiration (set to 100%) consisting of mitochondrial and non-mitochondrial respiration. Basal and maximal respiration was calculated by subtracting non-mitochondrial OCR. Respiratory reserve capacity was calculated as the difference between maximal and basal OCR. ATP-linked OCR was estimated as the difference between basal and rotenone/actinomycin A inhibited OCR.

### 2.8. Ferroptosis

A BrdU cell proliferation enzyme-linked immunosorbent assay (ELISA) kit (Calbiochem/Merck Biosciences, Darmstadt, Germany) was used to measure ferroptosis. Tumor cells were treated for 72 h with ART (10 µM) or ART combined with ferrostatin-1 (20 µM), a ferroptosis inhibitor. For more details see “Proliferation” (4.4) as described above.

### 2.9. Expression of Cell Cycle and Cell Death Regulating Proteins

To explore the expression and activity of cell cycle and cell death regulating proteins, Western blot analysis was performed. Tumor cell lysates (50 µg) were applied to 12 or 15% polyacrylamide gel and separated for 10 min at 80 V and 1 h at 150 V. The protein was then transferred to PVDF membranes (1 h, 100 V). After blocking with non-fat dry milk for 1 h, membranes were incubated overnight with the following monoclonal primary antibodies directed against cell cycle proteins: CDK1 (mouse IgG1, clone 1, dilution 1:5000, 34 kDa), CDK2 (mouse IgG2a, clone 55, dilution 1:5000, 33 kDa), CDK4 (mouse IgG1, clone 97, dilution 1:100, 34 kDa), Cyclin A (mouse IgG1, clone 25, dilution 1:500, 60 kDa), Cyclin B (mouse IgG1, clone 18, dilution 1:1000, 62 kDa), p27 (mouse IgG1, clone 57, dilution 1:1000, 27 kDa; all: BD Biosciences, Heidelberg, Germany) as well as phospho-CDK2 (rabbit IgG, Thr160, dilution 1:1000, 33 kDa), Cyclin D1 (rabbit IgG, clone 92G2, dilution 1:1000, 36 kDa), Cyclin E1 (rabbit IgG, clone D7T3U, dilution 1:1000, 48 kDa), p21 (rabbit IgG, clone 12D1, dilution 1:1000, 21 kDa; all: Cell Signaling, Frankfurt am Main, Germany). The following primary antibodies were employed to determine apoptosis proteins: p53 (rabbit IgG, clone 7F5, dilution 1:1000, 53 kDa), Caspase 3 (rabbit IgG, dilution 1:1000, 17, 35 kDa), Caspase 8 (rabbit IgG, clone D35G2, dilution 1:1000, 10, 57 kDa), PARP (total and cleaved, rabbit IgG, clone 46D11, dilution 1:1000, 116, 89kDa), Bcl-2 (rabbit IgG, clone D55G8, dilution 1:1000, 26 kDa), Bax (rabbit IgG, polyclonal, dilution 1:1000, 20 kDa; all: Cell Signaling, Frankfurt am Main, Germany), phospho-caspase 3 (rabbit IgG, Ser150, polyclonal, dilution 1:1000, 35 kDa) and phospho-caspase 8 (rabbit IgG, Tyr380, polyclonal, dilution 1:1000, 57 kDa; all: Invitrogen, Thermo Fisher Scientific, Darmstadt, Germany). For autophagy and ferroptosis related proteins the following primary antibodies were used: LC3B (non-lipidated LC3B-I and lipidated LC3B-II, rabbit IgG, polyclonal, dilution 1:1000, 14, 16 kDa; Cell Signaling, Frankfurt am Main, Germany), GPX4 (rabbit IgG, dilution 1:1000, 22 kDa, Abcam, Berlin, Germany). HRP-conjugated rabbit-anti-mouse IgG or goat-anti-rabbit IgG served as secondary antibodies (IgG, both: dilution 1:1000, Dako, Glosturp, Denmark).

The membranes were incubated with ECL detection reagent (AC2204, Azure Biosystems, Munich, Germany) to visualize the proteins with a Sapphire Imager (Azure Biosystems, Munich, Germany). The exposure time was adapted to the signal intensity (device-specific maximum, >65,000 = over-saturated). Only images with a maximum band intensity of below 65,000 were used for evaluation. All samples were normalized to total protein. To quantify total protein, all membranes were stained by Coomassie brilliant blue and measured by Sapphire Imager. AlphaView software (ProteinSimple, San Jose, CA, USA) was used to perform pixel density analysis of the protein bands. The ratio of protein intensity/whole protein intensity was calculated, and expressed in percentage, related to the untreated controls, set to 100%.

### 2.10. Statistics

All experiments were performed at least three times. The evaluation and generation of mean values, and normalization in percent were done by Microsoft Excel. Statistical significance was calculated with the GraphPad Prism 7.0 (GraphPad Software Inc, San Diego, CA, USA) two-way ANOVA test. Differences were considered statistically significant at *p* ≤ 0.05.

## 3. Results

### 3.1. ART Induced Significant Growth Inhibition of Parental and Cisplatin-Resistant Cells

**Figure 1:**
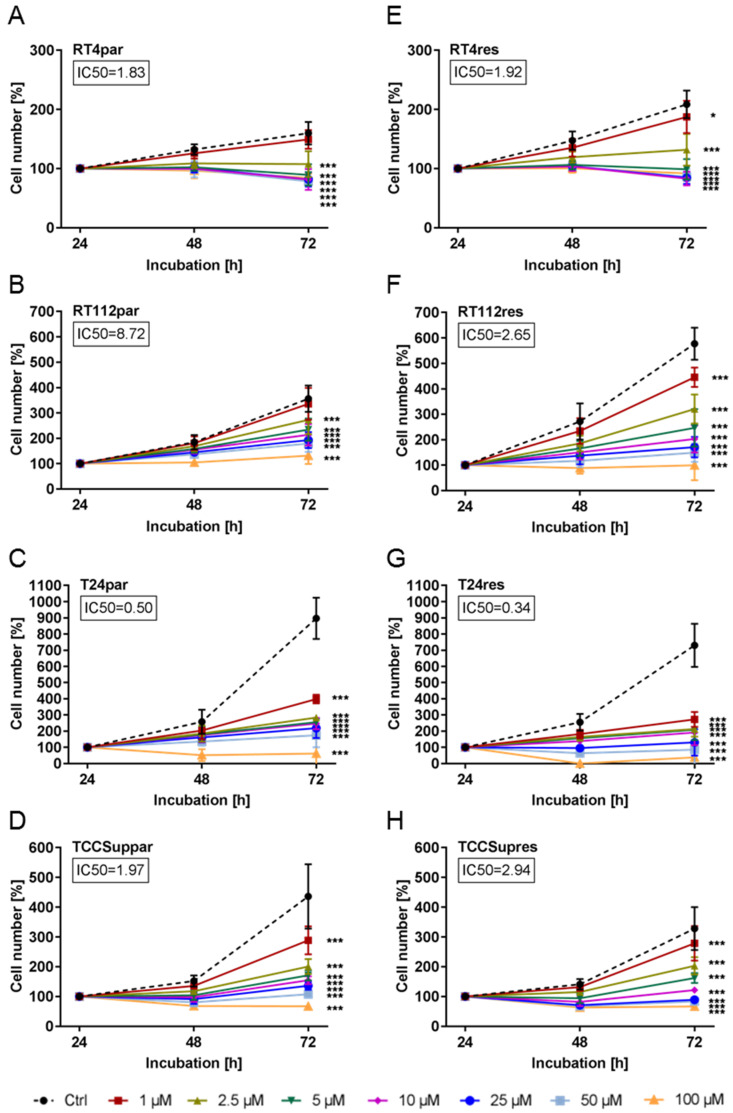
Tumor cell growth: Tumor cell growth (MTT-test) of parental (par) and cisplatin-resistant (res) RT4 (**A**,**E**), RT112 (**B**,**F**), T24 (**C**,**G**), and TCCSup (**D**,**H**) bladder cancer cells after 24, 48, and 72 h treatment with ascending concentrations of ART (1–100 µM). Cell number set to 100% after 24 h incubation. The half-maximal inhibitory concentration (IC50) of ART in µM after 72 h treatment is specified. Error bars indicate standard deviation (*SD*). Significant difference to untreated control: * *p* ≤ 0.05, *** *p* ≤ 0.001. n = 5.

Compared to their parental (par) counterparts, cell growth of cisplatin-resistant (res) RT4 (RT4res) and RT112 (RT112res) cells was enhanced (Figure 1A,B,E,F), whereas cisplatin-resistant T24 (T24res) and TCCSup (TCCSupres) cells grew to a lesser extent than their parental counterparts (Figure 1C,D,G,H). Exposure to ART (1–100 µM) resulted in a time- and dose-dependent significant growth inhibition in all parental and resistant BCa cells compared to untreated controls. The cytotoxic efficacy of ART was comparable between parental RT4 (RT4par) and RT4res cells (parental: IC50 = 1.83 µM, resistant: IC50 = 1.92 µM, Figure 1A,E). RT112res cells displayed a higher sensitivity to ART, compared to RT112par (parental: IC50 = 8.72 µM, resistant: IC50 = 2.65 µM, Figure 1B,F). In contrast, growth inhibition by ART in TCCSup was stronger in TCCSuppar, compared to the resistant cells (parental: IC50 = 1.97 µM, resistant: IC50 = 2.94 µM, Figure 1D,H). The most prominent growth inhibitory activity for ART was found in T24 cells (Figure 1C,G). IC50s at 0.50 µM in T24par and 0.34 µM in T24res cells (Figure 1C,G) also indicate a higher sensitivity to ART in T24res cells. Following the IC50 evaluation, two concentrations of ART were chosen for further experimentation, an efficient lower concentration (2.5 µM) and a higher concentration (10 µM), to determine whether ART’s mechanism of action may change at higher concentrations.

### 3.2. Proliferation of BCa Cells Significantly Decreased upon ART Application

**Figure 2:**
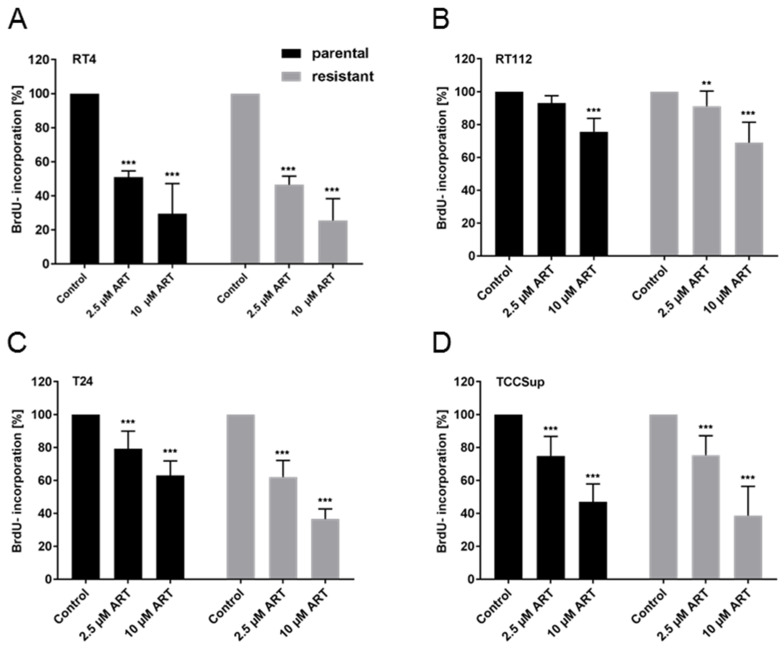
Cell proliferation: Tumor cell proliferation of parental (par) and cisplatin-resistant (res) RT4 (**A**), RT112 (**B**), T24 (**C**), and TCCSup (**D**) cells incubated for 48 h with ART (2.5 or 10 µM). Untreated controls were set to 100%. Error bars indicate standard deviation (*SD*). Significant difference to untreated control: ** *p* ≤ 0.01, *** *p* ≤ 0.001. n = 5.

In all cell lines, distinct tumor suppressive properties of ART on tumor cell proliferation were determined. Exposure to ART for 48 h resulted in a dose-dependent proliferation inhibition in RT4 (Figure 2A), RT112 (Figure 2B), T24 (Figure 2C), and TCCSup (Figure 2D) cells. As with cell growth, RT112res and T24res exhibited higher sensitivity to ART treatment in regard to proliferation than their corresponding parental counterparts, RT112par and T24par. In RT112res, exposure to 10 µM ART induced a 31.0% proliferation repression vs. 24.4% for RT112par (Figure 2B). In the T24res cells, the mean proliferation blockade in response to 10 µM ART was 63.4% vs. 37% in T24par (Figure 2C). The proliferation rate was diminished in TCCSuppar by 53% and in TCCSupres by 61.3% with 10 µM ART (Figure 2D). In RT4 the application of 2.5 µM ART resulted in a strong decrease in proliferation, contributing to a 51.4% proliferation reduction in RT4par and a 50.8% decrease in RT4res (Figure 2A). High-dosed ART (10 µM) caused further inhibition of proliferation in both RT4 cell lines. Since T24 and TCCSup cells displayed the highest sensitivity to ART in regard to growth and proliferation, these cell lines (T24par and T24res, TCCSuppar, and TCCSupres) were utilized for the following experiments.

### 3.3. ART Resulted in Cell Cycle Arrest and Elevation of Apoptotic Events

**Figure 3:**
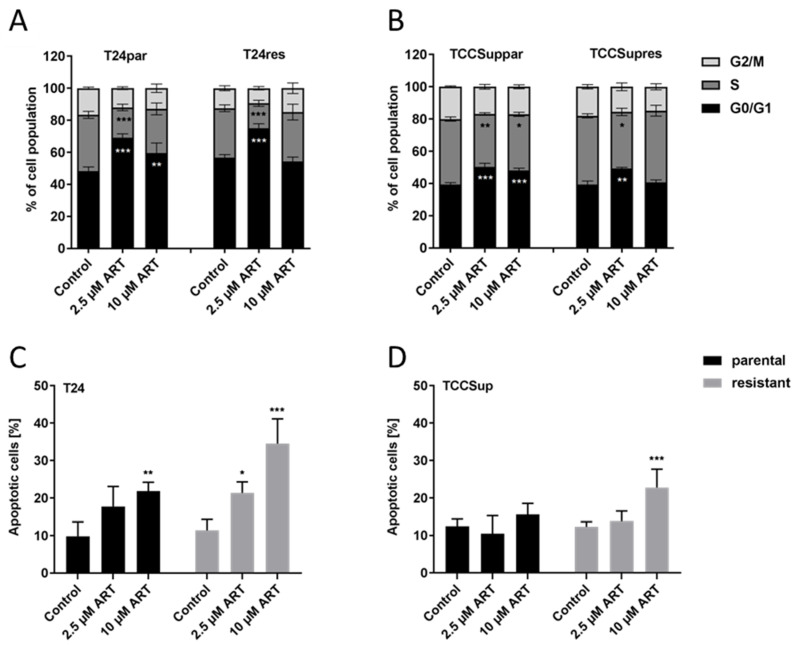
Distribution in the cell cycle phases and apoptotic events: Distribution in the cell cycle phases 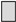 G2/, 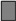 S, 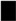 GO/G1) of parental and cisplatin-resistant T24 (**A**) and TCCSup (**B**) cells after 48 h (TCCSup) or 72 h (T24) exposure to ART. Percent of apoptotic events in parental and resistant T24 (**C**) and TCCSup (**D**) cells after 72 h treatment with ART (2.5 and 10 µM); comparison is to untreated controls. Error bars indicate standard deviation (*SD*). Significant difference to untreated control: * *p* ≤ 0.05, ** *p* ≤ 0.01, *** *p* ≤ 0.001. n = 4.

Reduced cell growth and proliferation in the BCa cells after exposure to ART was accompanied by cell cycle arrest and apoptosis induction in a concentration-dependent manner. A significant cell cycle arrest in the G0/G1 phase was detected in both parental and cisplatin-resistant T24 and TCCSup cells, especially in those treated with low-dosed ART (2.5 µM), compared to untreated controls (Figure 3A,B). The increase in G0/G1 phase cells was associated with a significant decrease in S phase cells. High-dosed ART (10 µM) induced less pronounced effects on cell cycle progression of parental T24 and TCCSup cells and no longer provoked an effect in their resistant counterparts.

In contrast, exposure of T24res and TCCSupres cells, as well as T24par cells, to 10 µM ART resulted in a significant elevation of apoptotic events (Figure 3C,D). Furthermore, treatment with 2.5 µM ART in T24res significantly, but to a lesser extent, induced apoptosis (Figure 3C). The number of necrotic cells was low and not significantly altered after exposure to any ART concentration (data not shown). Since parental and cisplatin-resistant T24 cells showed significant alteration after ART treatment for both cell cycle progression and apoptosis, the following investigation focused on that cell line.

### 3.4. ART Modulated Cell Cycle Regulating Proteins

**Figure 4:**
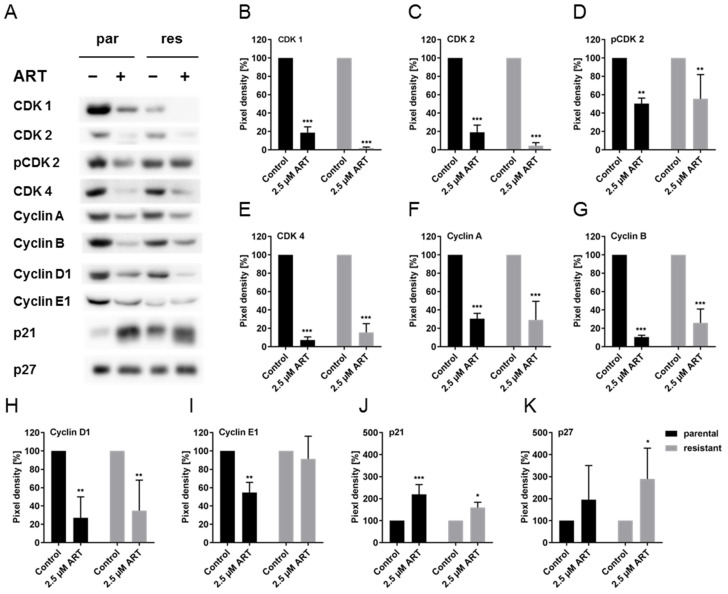
Protein expression profile of cell cycle regulating proteins: Representative Western blot analysis of cell cycle regulating proteins in parental (par) and cisplatin-resistant (res) T24 cells after 48 h exposure to 2.5 µM ART (**A**). Pixel density analysis of protein expression (**B**–**K**), compared to untreated controls (set to 100%), is illustrated. Each protein analysis was accompanied and normalized by a total protein control. Error bars indicate standard deviation (*SD*). Significant difference to untreated control: * *p* ≤ 0.05, ** *p* ≤ 0.01, *** *p* ≤ 0.001. n = 3. For detailed information regarding the Western blots see Supplementary Materials Figure S1.

Since impaired cell cycle progression with ART treatment was apparent, the expression of cell cycle regulating proteins, including cyclins, cyclin-dependent kinases (CDKs), and tumor suppressors, was analyzed. A significant down-regulation of proteins responsible for the transition from the G0/G1 to S phase was detected in response to ART. Notably, low expression levels of total CDK2 and pCDK2 were evident in T24par and T24res cells, compared to untreated controls (Figure 4A,C,D and Figure S1.2,S1.3). In good accordance was Cyclin E1, which associates with CDK2 to regulate progression from the G1 into S phase, significantly diminished in T24par after exposure to ART (Figure 4A,I and Figure S1.8). In both T24par and T24res cells, ART also significantly down-regulated Cyclin D1 and CDK4, required for entry into the G1 phase (Figure 4A,E,H, and Figure S1.4,S1.7). CDK1 and Cyclin A/B, essential during the late S phase and early M phase, were also down-regulated (Figure 4A,B,F,G, and Figure S1.1,S1.5,S1.6). ART induced a significant up-regulation of the tumor suppressor p21 in T24par and T24res cells (Figure 4A,J, and Figure S1.9). In T24res cells, the expression of p27 was also significantly increased with ART (Figure 4A,K and Figure S1.10).

### 3.5. ART Disrupted Mitochondrial Respiration

**Figure 5:**
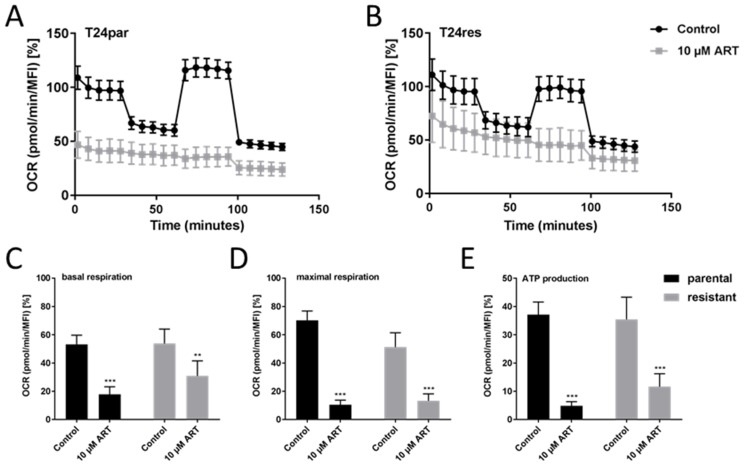
Mitochondrial respiration: Mitochondrial respiration in parental (**A**) and resistant (**B**) T24 cells, 24 h exposed to ART, compared to untreated controls. Data pertaining to the OCR were normalized to total basal respiration (set to 100%) consisting of mitochondrial and non-mitochondrial respiration. Extracted values for mitochondrial basal OCR (**C**) and maximal OCR (**D**) and ATP production (**E**) after ART treatment. MFI = mean fluorescent intensity. Error bars indicate standard deviation (*SD*). Significant difference to untreated control: ** *p* ≤ 0.01, *** *p* ≤ 0.001. n = 3.

To investigate whether ART provoked apoptosis associated with mitochondrial function, respiration was assessed. The oxygen consumption rate (OCR), as expressed through basal respiration, ATP production-coupled respiration, maximal and reserve capacities, and non-mitochondrial respiration, was impaired with ART treatment (Figure 5A,B). ART application resulted in significant systemic reduction of basal and maximal respiration in T24par and T24res cells, compared to untreated controls (Figure 5C,D). Moreover, spare respiratory capacity, reflecting cell ability to enhance respiration in response to physiological or pharmacological stress (maximal minus basal respiration), was completely abrogated by ART in T24par and T24res cells (Figure 5A,B). Diminished OCR was accompanied by reduced ATP production (Figure 5E). No change in extracellular acidification rate, indicating anaerobic glycolytic activity, was detected after exposure to ART in the BCa cells, and thus, no shift towards compensatory glycolysis was apparent (data not shown).

### 3.6. ART Inhibited DNA Damage Repair and Modulated Cell Death-Associated Proteins

**Figure 6:**
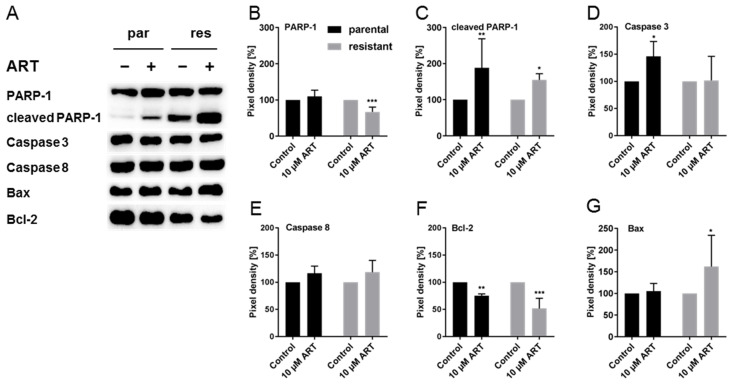
Apoptosisand DNA damage repair associated proteins: Representative Western blot analysis of DNA damage repair and cell death associated proteins in parental (par) and cisplatin-resistant (res) T24 cells after 24 h exposure to 10 µM ART (PARP-1 = non-cleaved PARP-1) (**A**). Pixel density analysis of the protein expression (**B**–**G**), compared to the untreated controls (set to 100%) is illustrated. Each protein analysis was normalized by a total protein staining control. Error bars indicate standard deviation (*SD*). Significant difference to untreated control: * *p* ≤ 0.05, ** *p* ≤ 0.01, *** *p* ≤ 0.001. n = 4. For detailed information regarding the Western blots see Figure S2.

Since cisplatin causes DNA damage, the expression and activity of poly(ADP-ribose) polymerase 1 (PARP-1), a nuclear enzyme involved in DNA damage repair and DNA stability, was analyzed in the T24 cell lines after ART application. In T24par cells, active PARP-1 (cleaved PARP-1) was significantly enhanced after exposure to ART, compared to the untreated control, whereas expression of non-cleaved PARP-1 was not altered (Figure 6A–C and Figure S2.1). In T24res cells, ART significantly reduced non-cleaved PARP-1 and increased levels of cleaved PARP-1 (Figure 6B,C and Figure S2.1), indicating high PARP-1 activity. The expression of total caspase-3 was significantly enhanced after 24 h exposure to 10 µM ART in T24par cells, but remained unaltered in the T24res cells (Figure 6A,D and Figure S2.2). Expression of caspase-8 did not change after 24 h ART treatment (Figure 6A,E and Figure S2.3). No signal was detected for either cleaved-caspase-3 or phospho-caspase-3 and -8.

Inasmuch as the higher concentration of ART resulted in an elevation of apoptotic events and ART primarily accumulates in mitochondria [39], expression of the key proteins Bcl-2 and Bax, involved in mitochondria-associated apoptosis, was evaluated. Bcl-2, an apoptosis inhibitor, was significantly down-regulated by ART in both T24par and T24res cells, whereas the expression of Bax, an apoptosis activator, was elevated in the T24res cells, compared to their respective untreated controls (Figure 6A,F,G and Figure S2.4,S2.5).

**Figure 7:**
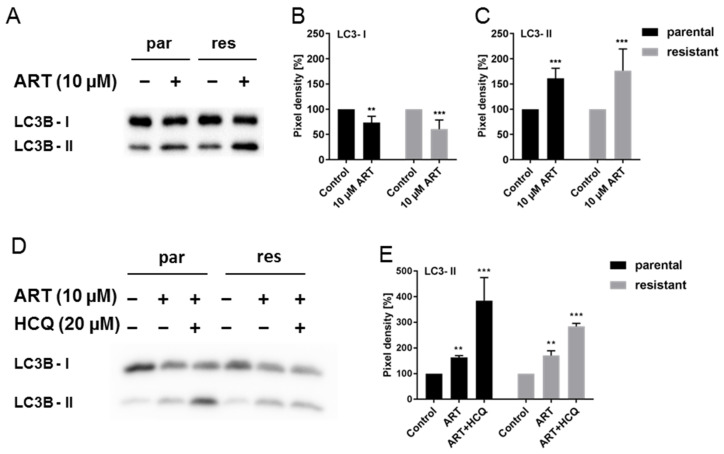
Autophagy: Expression of LC3B-I and LC3B-II in parental (par) and cisplatin-resistant (res) T24 cells after 24 h exposure to 10 µM ART alone (**A**) or in combination with 20 µM HCQ (**D**). A representative Western blot of n = 4 is shown. Each protein analysis was normalized by a total protein staining control. Pixel density analysis of the protein expression (**B**,**C**,**E**), compared to the untreated controls (set to 100%) is illustrated. Error bars indicate standard deviation (*SD*). Significant difference to untreated control: ** *p* ≤ 0.01, *** *p* ≤ 0.001. n = 4. For detailed information regarding the Western blots see Figure S3.

In addition to apoptotic markers, expression of the autophagy markers LC3B-I and LC3B-II was evaluated. ART resulted in a significant increase in LC3B-II, an autophagosome membrane bound form of the LC3B protein, in both T24par and T24res cells, compared to the untreated controls (Figure 7A,C and Figure S3.1). At the same time, expression of LC3B-I was diminished (Figure 7A,B and Figure S3.1), indicating dynamic autophagic activity (also called autophagic flux). To validate autophagic flux in the presence of ART, T24 cells were exposed to a combination of ART (10 µM) and hydroxycloroquine (HCQ) (20 µM), a lysosomotropic agent that blocks late autophagy by disrupting autophagosome and lysosome fusion [40]. A significant elevation of LC3B-II levels was observed in the presence of HCQ, compared to ART application alone, in both parental and resistant cells (Figure 7D,E and Figure S3.2), caused by an accumulation of newly formed autophagosomes that cannot be degraded by lysosomes.

### 3.7. ART Did Not Induce Ferroptosis in BCa Cells

**Figure 8:**
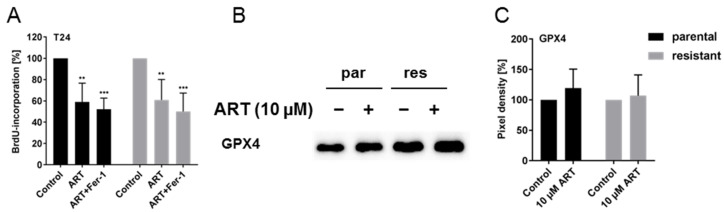
Ferroptosis: Cell proliferation of T24par and T24res after 72 h incubation with 10 µM ART alone or plus 20 µM ferrostatin-1 (Fer-1), a ferroptosis inhibitor, in percent (**A**). Untreated controls were set to 100%. Error bars indicate standard deviation (*SD*). Significant difference to untreated control: ** *p* ≤ 0.01, *** *p* ≤ 0.001. n = 3. Representative Western blot of GPX4 expression in T24 cells after 24 h exposure to 10 µM ART (**B**). par = parental, res = resistant. Pixel density analysis of the GPX4 expression (**C**), compared to untreated controls (set to 100%) is illustrated. Each protein analysis was normalized by a total protein staining control. Error bars indicate standard deviation (*SD*). n = 4. For detailed information regarding the Western blots see Figure S4.

Because ART induced a caspase-independent permeabilization of the outer mitochondrial membrane, a possible role of ferroptosis, a ROS- and iron-dependent programmed cell death, was evaluated in parental and resistant bladder cancer cells. Treatment of T24 cell lines with ART (10 µM) and ferrostatin-1 (20 µM), a ferroptosis inhibitor, did not reverse proliferation inhibition seen under ART treatment alone, indicating a lack of ferroptotic events (Figure 8A). Accordingly, after ART exposure, T24par and T24res cells revealed no alteration in the expression of GPX4, an antioxidant activated in the presence of ROS during ferroptosis (Figure 8B,C and Figure S4).

## 4. Discussion

Despite progress in cisplatin-based chemotherapy for advanced BCa, disease relapse remains a challenge, in part due to adaptive mechanisms of the tumor cells to cisplatin-induced DNA damage. This leads to drug resistance and often results in therapeutic failure. Thus, innovative therapy strategies are urgently required. Recent studies have shown that the anti-proliferative activity of ART on solid tumors is achieved through oxidative stress and compromising DNA repair mechanisms, making ART a promising drug to oppose tumor cell adaptation to cisplatin.

In the present study, an anti-cancer activity of ART in different histological types of parental and cisplatin-resistant BCa cells was ascertained. Cell growth and proliferation in both parental and cisplatin-resistant BCa cells were potently inhibited by ART in a time- and dose-dependent manner. Overall, the growth inhibitory effect of ART was more pronounced in the fast growing RT112, T24 and TCCSup cell lines, compared to the slow growing RT4 cells. The ART concentrations affecting the BCa cells in vitro are clinically relevant, since concentrations in this range have been used to successfully treat malaria patients with ART [41]. Consistent with the findings presented here, ART exhibited a dose-dependent anti-proliferative activity in neuroblastoma [14], B-cell lymphoma [42], liver [43], colorectal [32], breast [44], tongue [33,45], cervical [46], ovarian [47,48], and esophageal [49] cancers. In a small panel of therapy-sensitive BCa cells ART has specifically targeted bladder cancer cells but not normal urothelial cells in vitro [34]. Moreover, ART has effectively reduced tumor cell growth of BCa in an orthotopic rat model [34]. In a rat model of colorectal cancer, ART has inhibited pro-inflammatory signaling and reduced cell proliferation [50,51].

Several investigators have shown that ART restricted cancer cell growth through induction of oxidative DNA damage [13,52], induction of apoptosis [14,29,53,54] and cell cycle arrest [48]. In the current study the exposure to low-dosed ART (2.5 µM) resulted in a G0/G1 phase arrest, accompanied by a decreased S phase in parental and cisplatin-resistant BCa cells. Apoptotic events were not detected at this ART concentration except in T24res, indicating that cell growth inhibition at lower concentrations mainly occurred due to cell cycle arrest. In line with this, treatment with 2.5 µM ART resulted in distinctive modulation of cell cycle regulating proteins. The expression of the cell cycle activating proteins CDK1, CDK2, pCDK2, CDK4, cyclin A, B, D1, and E1 was diminished, whereas the cell cycle inhibiting proteins p21, and to a lesser extent p27, increased in parental and cisplatin-resistant BCa cells in response to ART (2.5 µM). Accordingly, ART caused a G0/G1 phase arrest in vitro and in vivo in esophageal cancer by down-regulating CDC25A, a CDK2 activator, thereby compromising entry into the S phase [49]. ART restricted proliferation of lung cancer cells in the G0/G1 phase by decreasing Cyclin D1, Cyclin E, CDK2, and CDK4 expression [55]. Furthermore, ART down-regulated the expression of CDKs 2 and 4, and Cyclins D1 and E in breast cancer [56] and enhanced the expression of the cell cycle negative regulators, p21 and 27, in gastric cancer [57].

Recent studies showed that low expression of p21 was strongly correlated with tumor progression and a poor prognosis in esophageal [58], ovarian [59], and bladder [60] carcinomas. Moreover, up-regulation and gain-of-function manipulation of p21 has been proposed as a promising strategy to inhibit bladder tumor cell growth [60,61]. Thus, impaired G1/S transition in parental and resistant BCa cells can, at least partially, be attributed to elevated expression of the cell cycle inhibiting protein, p21, and to a lesser extent p27. However, further studies are needed to clarify the relation between sustained DNA damage, G0/G1 arrest, and p21 overexpression in ART-treated bladder cancer.

Treatment with high-dosed ART (10 µM) triggered apoptosis and displayed no or minor changes in cell cycle progression in the present investigation. In ovarian cancer cells, dose-dependent inhibition of proliferation by ART caused cell cycle arrest in the G0/G1 phase at a lower concentration and elongation of the G2/M phase at a higher concentration depending on the ROS level [48]. The cytotoxic effect of ART was evidenced by an accumulation of cleaved PARP-1 in the parental and cisplatin-resistant BCa cells, indicating augmented DNA damage induced by the combined application of cisplatin and ART. This may contribute to a critical amount of DNA damage, abrogating apoptosis circumvention and re-sensitizing the cells towards cisplatin. A similar effect of ART was also reported for neuroblastoma [14], colon cancer [32], breast cancer [33], and T-cell leukemia [29]. The synergistic action of ART and cisplatin induced DNA double strand breaks and reduced the clonogenic activity in ovarian cancer cells [12]. Furthermore, ART induced apoptosis with enhanced PARP-1 and caspase-3 activity in therapy-sensitive T24 cells [34,62]. Consistently, the expression of total caspase-3 significantly increased in parental but not in cisplatin-resistant BCa cells in the current investigation. However, neither activation of caspase-3 nor of caspase-8 was detectable in response to ART treatment. Instead, a significant decrease of anti-apoptotic Bcl-2 and an increase of pro-apoptotic Bax in the BCa cells were detected, indicating that ART might function partly by inducing an intrinsic mitochondria-mediated death pathway. Moreover, in colon cancer in vitro and in vivo, ART induced apoptosis by concomitantly increasing the expression of PARP-1 and Bax and decreasing Bcl-2 [32]. In gastric cancer cells, ART-mediated apoptosis was associated with down-regulation of CDC25A and Bcl-2 and up-regulation of Bax accompanied by reduced mitochondrial membrane potential levels [63]. Bcl-2 family proteins regulate DNA damage-induced apoptosis by controlling mitochondrial outer membrane permeabilization (MOMP) and the release of mitochondrial cytochrome C. Importantly, MOMP often leads to cell death, irrespective of caspase activity [64]. It has been reported that mitochondria are a primary target of cisplatin-induced oxidative stress [65] and that ART mainly accumulated in the mitochondria [39]. Both result in the extenuation of mitochondrial outer membrane potential and progressive loss of mitochondrial function. In the present investigation, mitochondrial dysfunction in response to ART was confirmed in both parental and cisplatin-resistant BCa cells. We found extensively impaired basal and maximal mitochondrial respiration, completely abrogated reserved respiratory capacity in therapy-sensitive and therapy-resistant cells, as well as eradicating ATP production. This strongly indicates that ART affected mitochondrial energy production, thereby drastically diminishing mitochondrial respiratory activity and the fitness of bladder cancer cells. Consistent with these findings, ART induced mitochondrial dysfunction via inhibition of mitochondrial respiration and decreasing ATP levels in nasopharyngeal carcinoma cells [66]. Similarly, artemisinin diminished oxygen consumption in hepatocellular carcinoma [67]. Caspase-independent BCa cell death might partly be explained by permeabilization of mitochondria in response to ART-related physiological or pharmacological stress stimuli [68].

Ferroptosis, another caspase-independent but ROS- and iron-dependent cell death, could theoretically have contributed to the ART-mediated death of the BCa cells. A recent study demonstrated that ART selectively induced ferroptosis in pancreatic cancer cells carrying a mutation in the KRAS gene. This process was blocked by the ferroptosis inhibitor ferrostatin-1 [69]. In addition, ART eliminated head and neck cancer cells via activation of iron-dependent ferroptosis by ROS-accumulation [16]. However, in the present investigation, neither ferroptosis activation nor GPX4 expression alteration, relevant for antioxidant activity, was detected as a result of ART application in either T24par or T24res BCa cells.

ART induced cellular autophagic flux in parental and cisplatin-resistant bladder cancer cells, as evidenced by the accumulation of autophagosome membrane bound LC3B-II protein. Application of the lysosomal inhibitor, HCQ [40], resulted in further elevated LC3B-II, confirming that autophagic activity was up-regulated upon exposure to ART. HCQ disrupts the autophagosome and lysosome fusion, resulting in an accumulation of newly formed autophagosomes [40] and therewith LC3B-II. In line with our observation, Zhou and colleagues demonstrated that ART triggers autophagy-mediated apoptotic cell death in therapy-sensitive BCa validated by elevated LC3B-II and cleaved caspase-3 expression [62]. The authors found that simultaneous activation of the energy-sensing kinase AMPK and inactivation of the nutrient-sensing kinase mTOR control autophagy induction via ULK1 after treatment with ART. In colon cancer cells, ART treatment caused autophagy activation, evident by increasing LC3B-II and beclin-1 light chain levels and elevated numbers of autophagosomes [32]. Here, blocking autophagy by HCQ led to enhanced apoptosis, indicating that ART prevented apoptosis in colon cancer [32]. These investigators therefore concluded that ART may hold potential for treating colon cancer, but may be more effective in combination with autophagy inhibitors. This mechanism seems to be unlikely for BCa cells and some other tumor entities, where autophagy is an indicator of drug response to chemotherapy. In good accordance with the present investigation, ART induced autophagy and simultaneously diminished cell growth by cell cycle arrest in ovarian cancer cells [35]. Moreover, ART triggered cellular autophagy and exhibited anti-proliferative activity in surgery-induced knee arthrofibrosis in vitro and in vivo [36]. Furthermore, temserolimus induced autophagy and thereby enhanced the sensitivity of BCa cell lines to gemcitabine and cisplatin [70]. Additionally, curcumin re-sensitized gefitinib-resistant small-cell lung cancer cells to gefitinib through an autophagy-dependent synergism [71]. ART also induced mitophagy, a selective type of autophagy targeting damaged mitochondria, which alters the cellular redox status [39]. Since ART impaired mitochondrial activity and induced autophagy, activated mitophagy could also be a reason for mitochondrial deactivation and caspase-independent cell death in bladder cancer cells.

## 5. Conclusions

ART significantly inhibited tumor cell growth and proliferation in a time- and dose-dependent manner in cisplatin-sensitive, and more importantly, cisplatin-resistant, BCa cells. At a low concentration, ART (2.5 µM) triggered G0/G1 cell cycle arrest by blocking G1/S transition. At a higher concentration, ART (10 µM) provoked mitochondrial malfunction, activated autophagy, and thus mediated apoptotic cell death in parental and cisplatin-resistant bladder cancer cells. Therefore, ART may hold promise as an additive to improve the cisplatin-based treatment of patients with advanced BCa and/or cisplatin-resistant BCa. However, further investigations are necessary to verify our postulate and in vivo studies need to clarify whether ART shows similar anti-tumor effects under physical conditions in parental and cisplatin-resistant BCa.

## Supplementary Materials

The following are available online at https://www.mdpi.com/2073-4409/9/12/2643/s1, Figure S1: Detailed information about Figure 4-Protein expression profile of cell cycle regulating proteins in parental and cisplatin-resistant T24 cells after 48 h exposure to ART, Figure S2: Detailed information about Figure 6-Apoptosis associated proteins in parental and cisplatin-resistant T24 cells after 24 h exposure to ART, Figure S3: Detailed information about Figure 7-Autophagy: Expression of LC3B-I and LC3B-II in parental and cisplatin-resistant T24 cells after 24 h exposure to ART alone or in combination with HCQ, Figure S4: Detailed information about Figure 8-Ferroptosis: GPX4 expression in parental and cisplatin-resistant T24 cells after 24 h exposure to ART.
