# Supplementary material for: Artesunate Impairs Growth in Cisplatin-Resistant Bladder Cancer Cells by Cell Cycle Arrest, Apoptosis and Autophagy Induction
Source: Cells. 2020 Dec 9;9(12):2643. doi: 10.3390/cells9122643 (PMC7763932; doi:10.3390/cells9122643)
Supplement: Supplementary file 1 [file cells-09-02643-s001.pdf]

Cell cycle regulating proteins

Figure S1.1 (a) T24par and T24res CDK1 (34kDa)
(b) Coomassie Brilliant Blue

(a) (b)

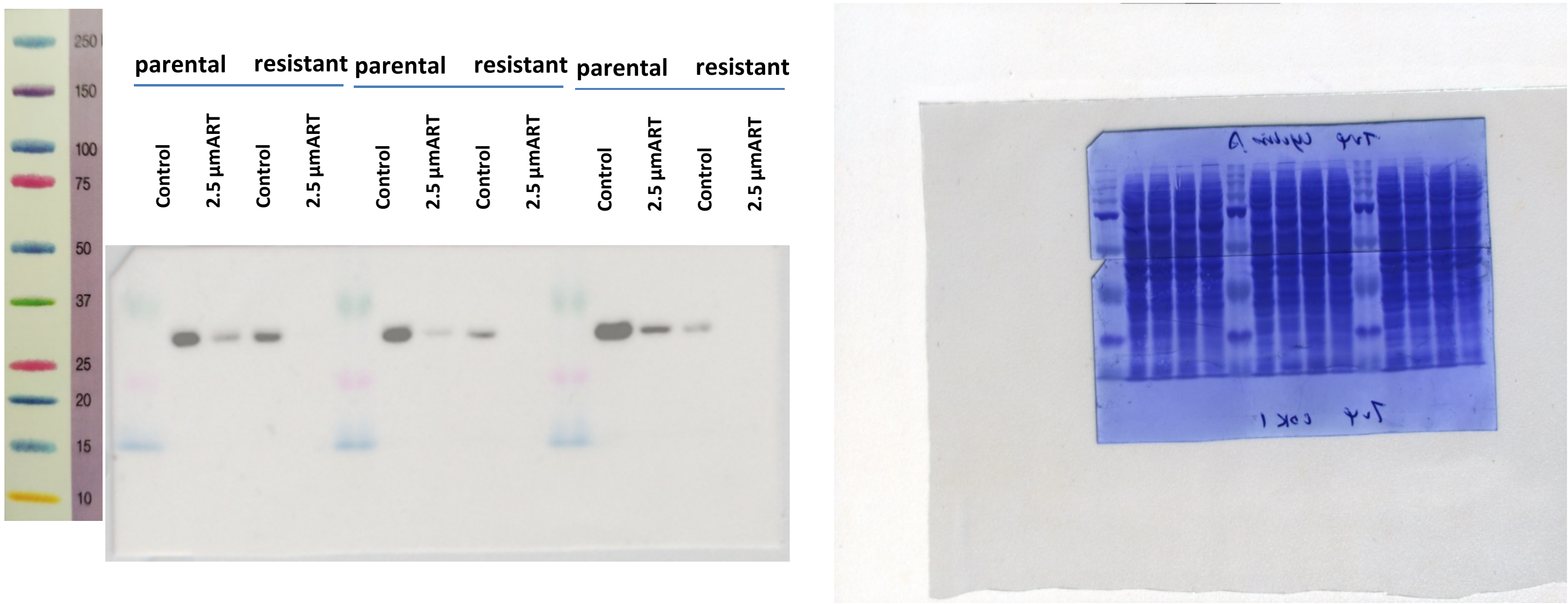

Figure S1.1: Detailed information about Figure 4 - Protein expression profile of cell cycle regulating proteins in parental and resistant T24 cells. Protein expression of CDK1 (a), corresponding Coomassie blue staining of total protein (b).

Figure S1.2 (a) T24par and T24res CDK2 (33 kDa)
(b) Coomassie Brilliant Blue

(a)

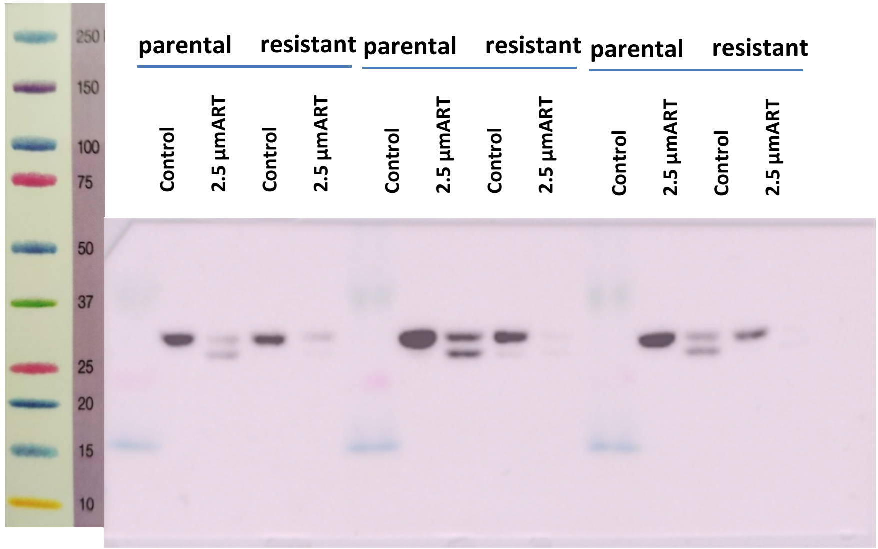

(b)

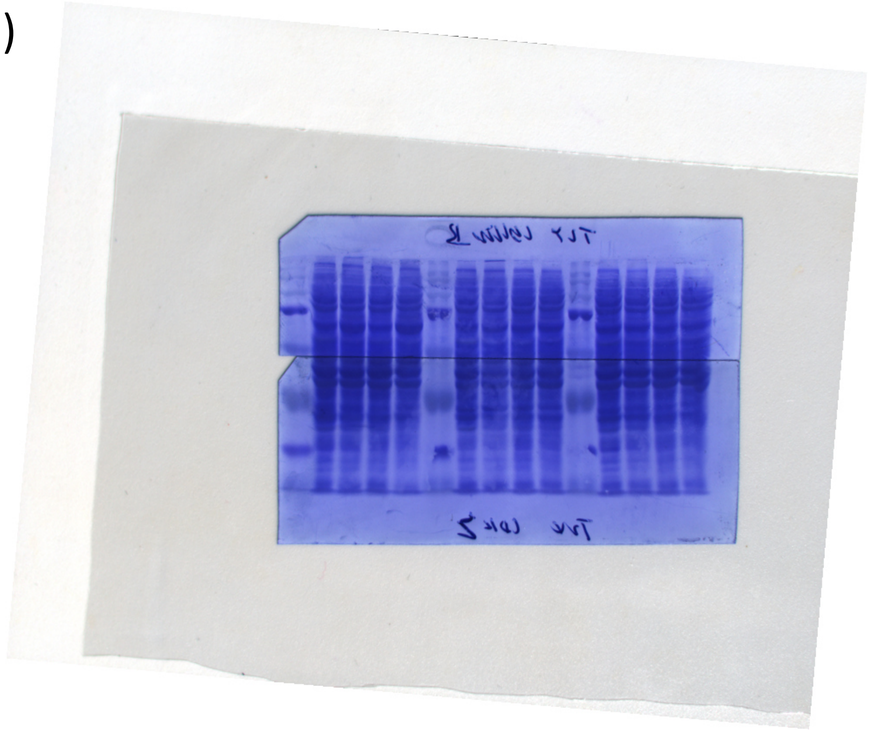

Figure S1.2: Detailed information about Figure 4 - Protein expression profile of cell cycle regulating proteins in parental and resistant T24 cells. Protein expression of CDK2 (a), corresponding Coomassie blue staining of total protein (b).

Figure S1.3 (a) T24par and T24res pCDK2 (33 kDa)
(b) Coomassie Brilliant Blue

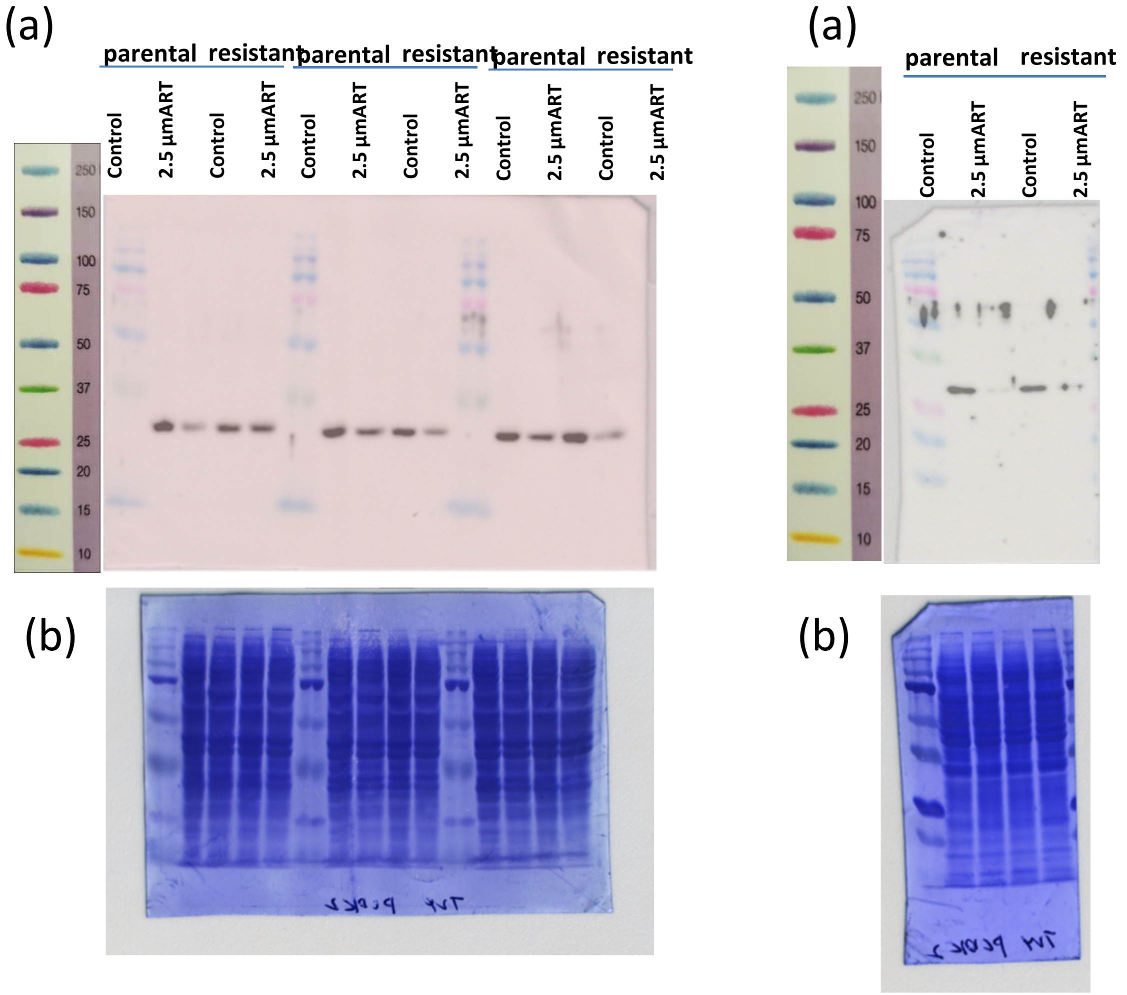

Figure S1.3: Detailed information about Figure 4 - Protein expression profile of cell cycle regulating proteins in parental and resistant T24 cells. Protein expression of pCDK2 (a), corresponding Coomassie blue staining of total protein (b).

Figure S1.4 (a) T24par and T24res CDK4 (34 kDa)
(b) Coomassie Brilliant Blue

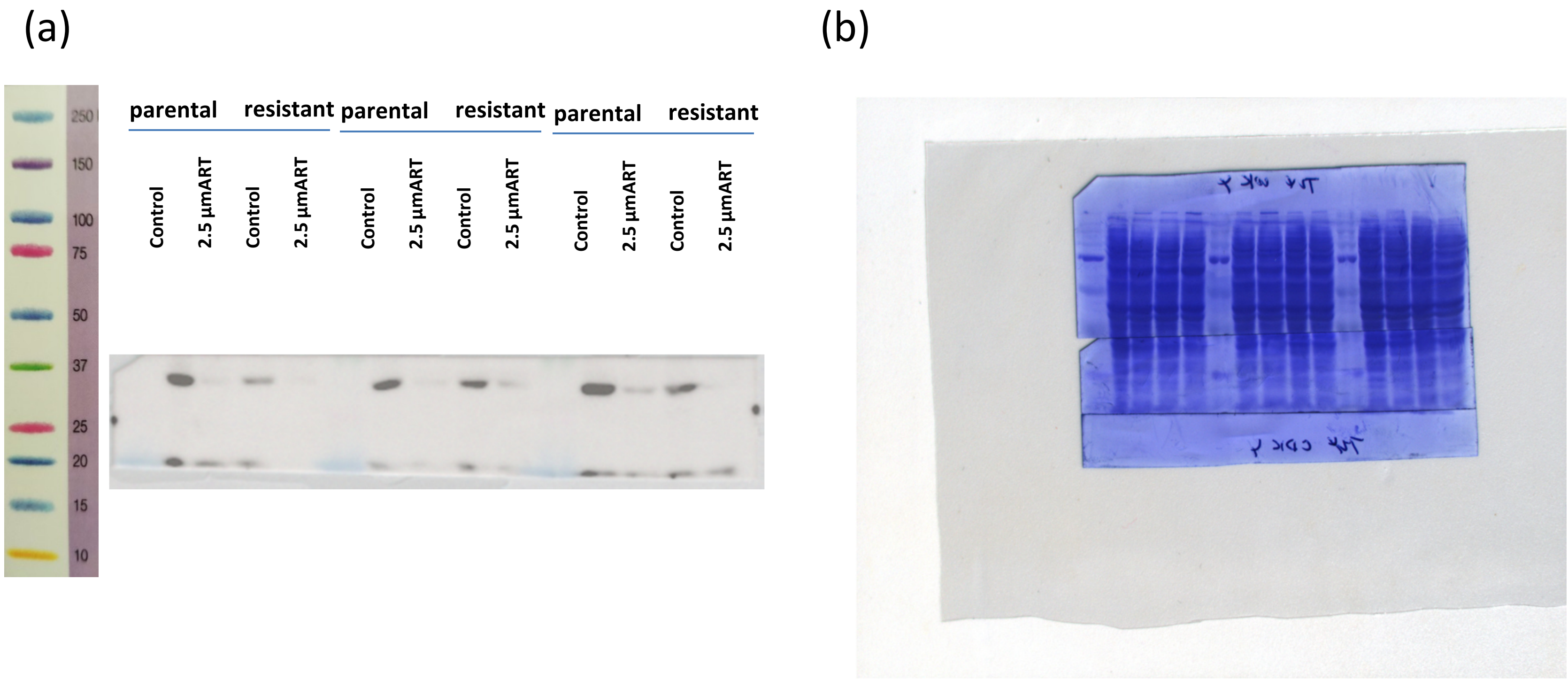

Figure S1.4: Detailed information about Figure 4 - Protein expression profile of cell cycle regulating proteins in parental and resistant T24 cells. Protein expression of CDK4 (a), corresponding Coomassie blue staining of total protein (b).

Figure S1.5 (a) T24par and T24res Cyclin A (60 kDa)
(b) Coomassie Brilliant Blue

(a)

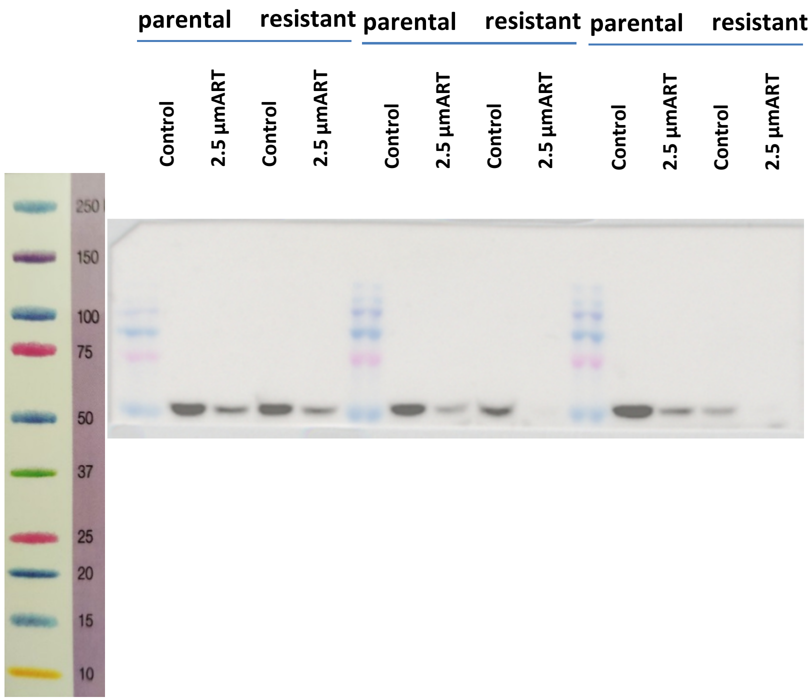

(b)

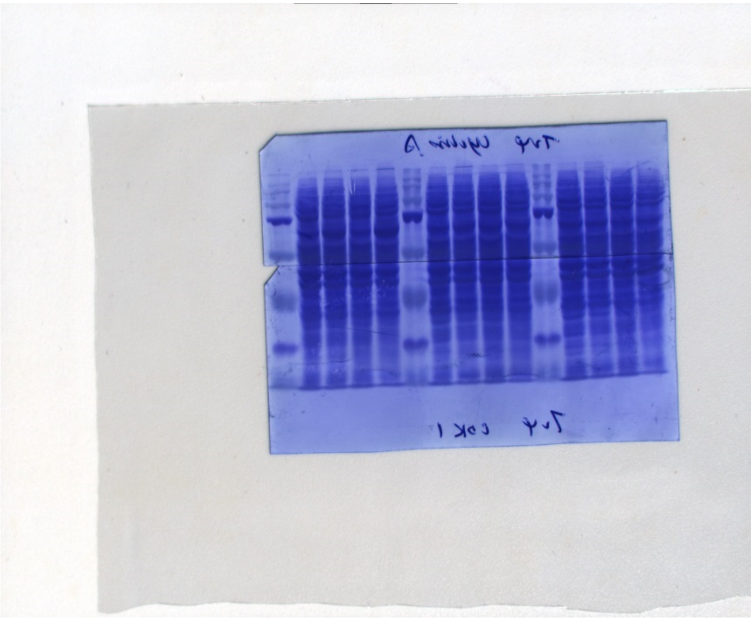

Figure S1.5: Detailed information about Figure 4 - Protein expression profile of cell cycle regulating proteins in parental and resistant T24 cells. Protein expression of Cyclin A (a), corresponding Coomassie blue staining of total protein (b).

Figure S1.6 (a) T24par and T24res Cyclin B (62 kDa)
(b) Coomassie Brilliant Blue

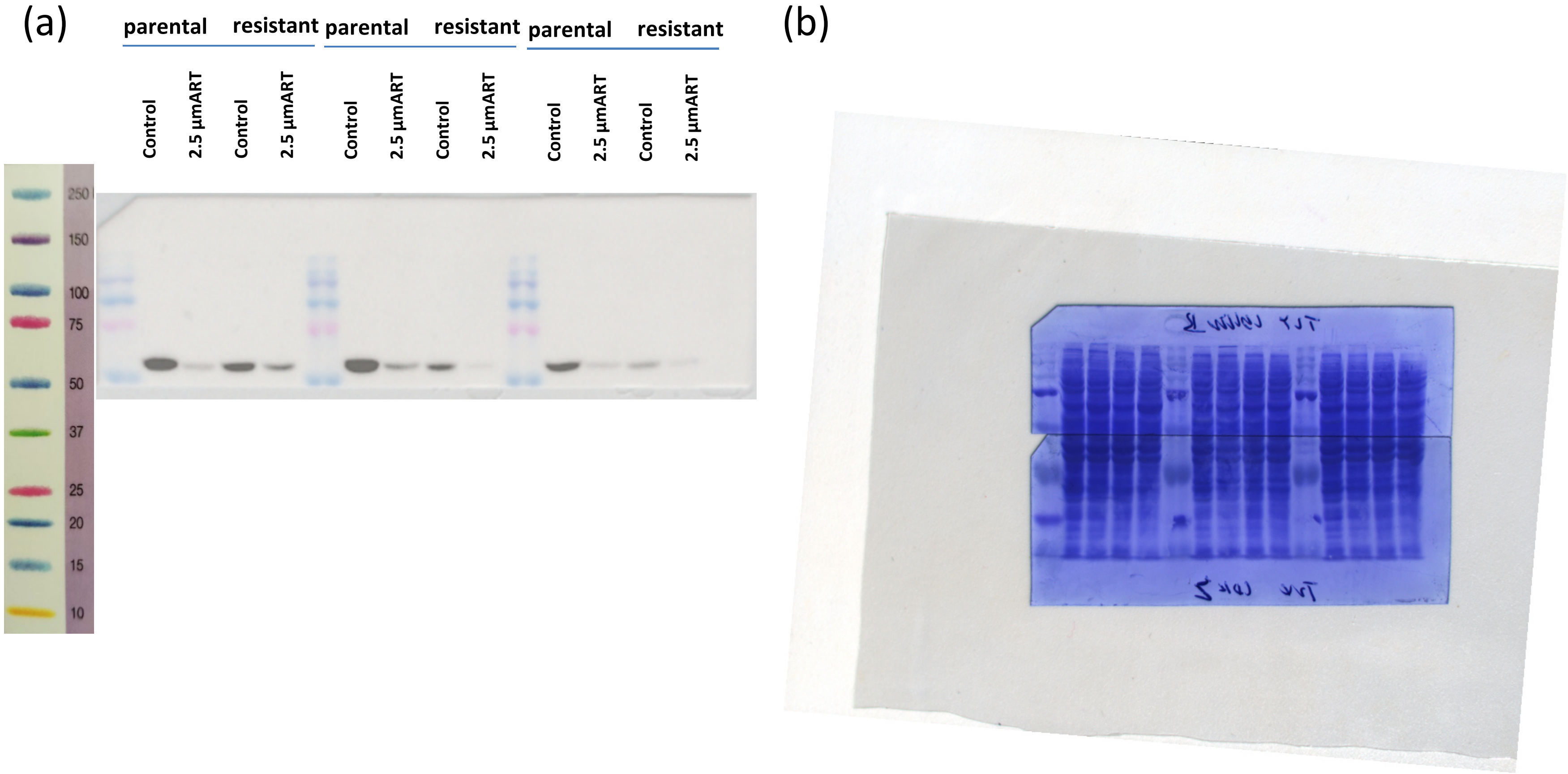

Figure S1.6: Detailed information about Figure 4 - Protein expression profile of cell cycle regulating proteins in parental and resistant T24 cells. Protein expression of Cyclin B (a), corresponding Coomassie blue staining of total protein (b).

Figure S1.7 (a) T24par and T24res Cyclin D1 (34 kDa)
(b) Coomassie Brilliant Blue

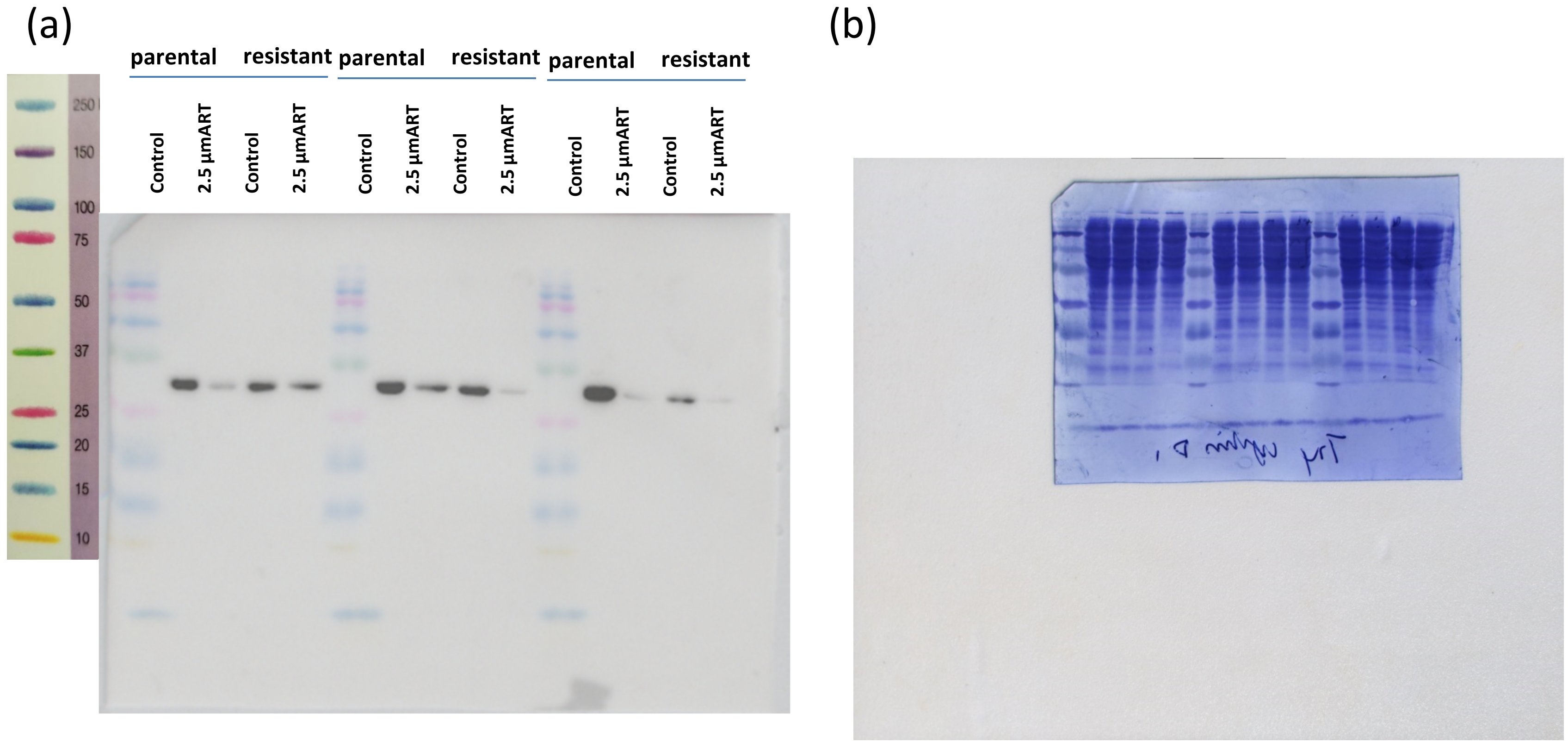

Figure S1.7: Detailed information about Figure 4 - Protein expression profile of cell cycle regulating proteins in parental and resistant T24 cells. Protein expression of Cyclin D1 (a), corresponding Coomassie blue staining of total protein (b).

Figure S1.8 (a) T24par and T24res Cyclin E1 (48 kDa)
(b) Coomassie Brilliant Blue

(a) (b)

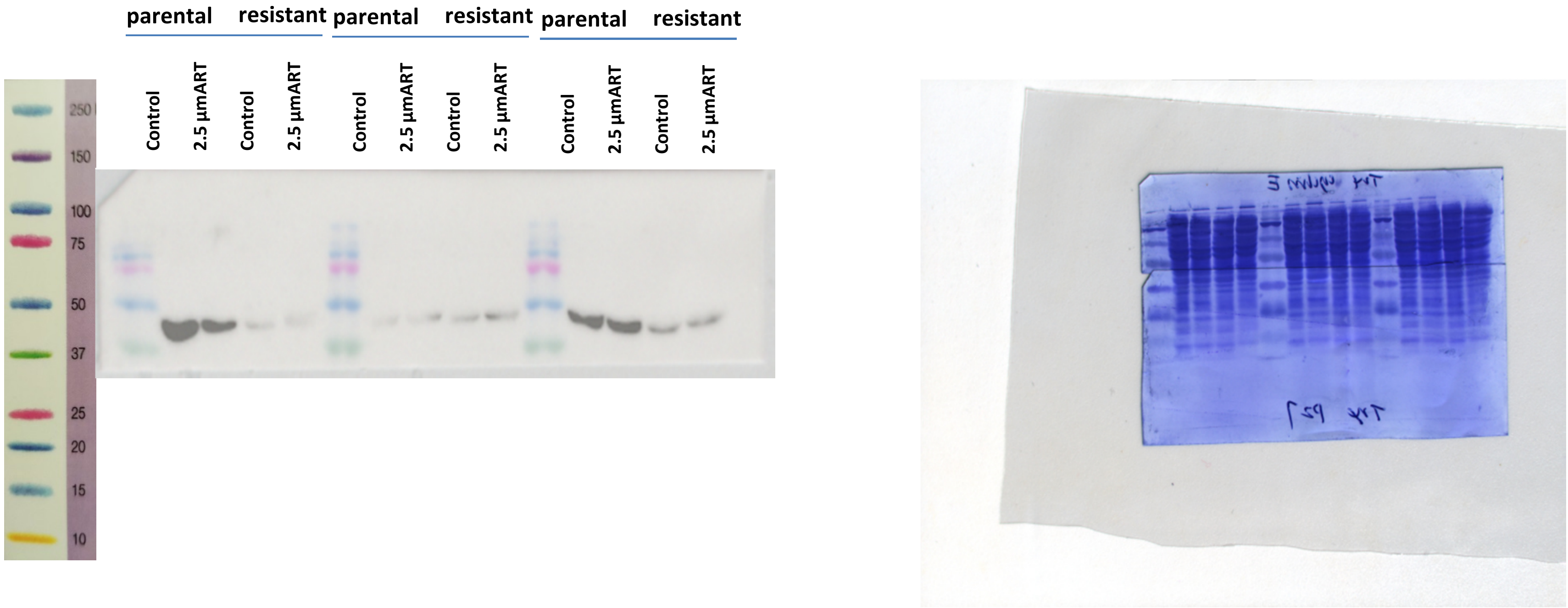

Figure S1.8: Detailed information about Figure 4 - Protein expression profile of cell cycle regulating proteins in parental and resistant T24 cells. Protein expression of Cyclin E (a), corresponding Coomassie blue staining of total protein (b).

Figure S1.9 (a) T24par and T24res p21 (21 kDa)
(b) Coomassie Brilliant Blue

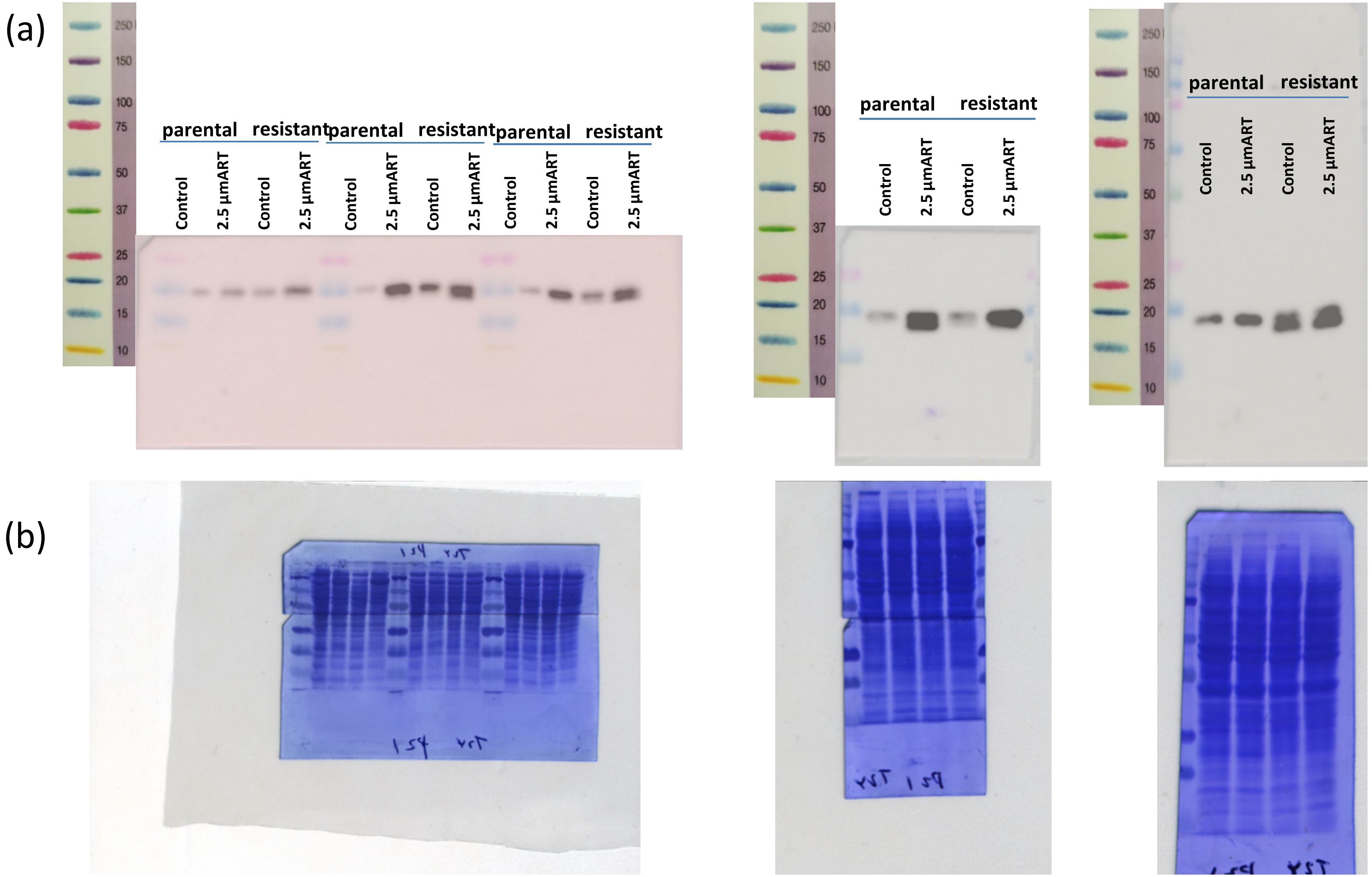

Figure S1.9: Detailed information about Figure 4 - Protein expression profile of cell cycle regulating proteins in parental and resistant T24 cells. Protein expression of p21 (a), corresponding Coomassie blue staining of total protein (b).

Figure S1.10 (a) T24par and T24res p27 (27 kDa)
(b) Coomassie Brilliant Blue

(a)

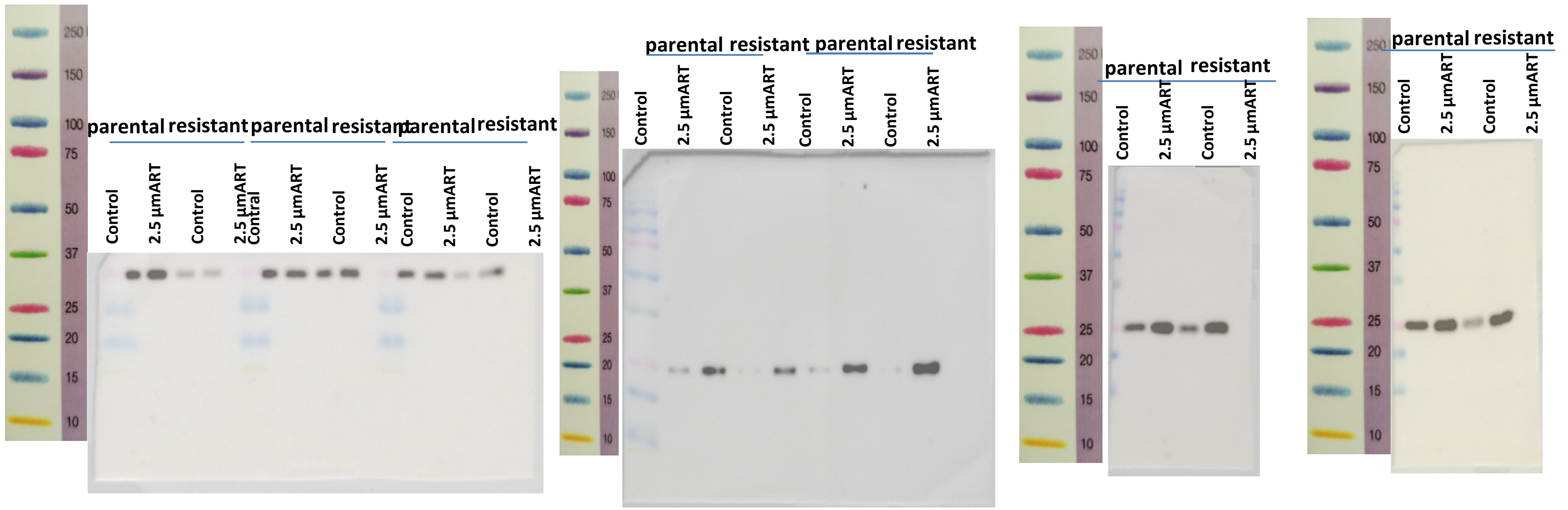

(b)

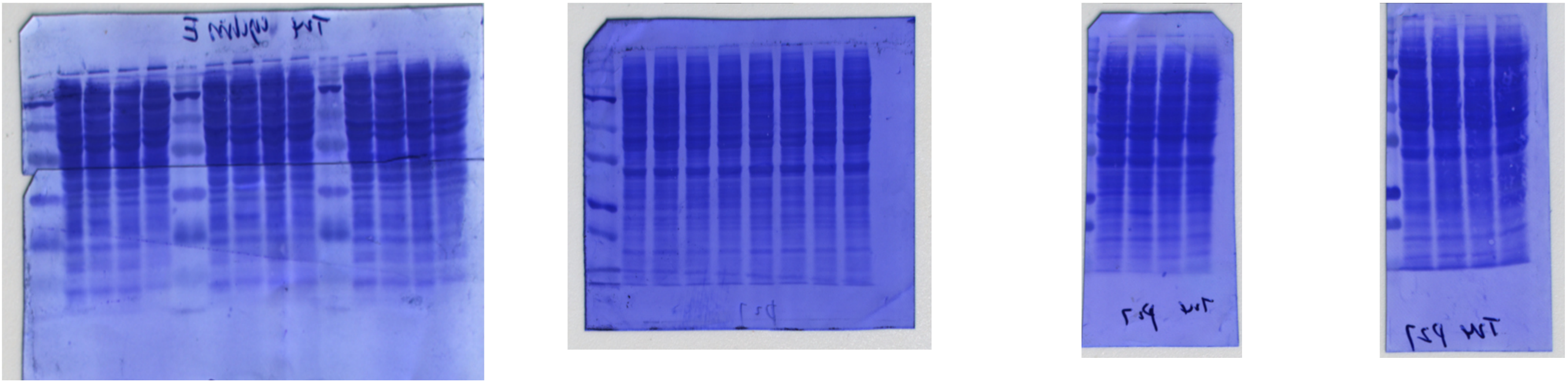

Figure S1.10: Detailed information about Figure 4 - Protein expression profile of cell cycle regulating proteins in parental and resistant T24 cells. Protein expression of p27 (a), corresponding Coomassie blue staining of total protein (b).

Cell death related proteins

Figure S2.1 (a) T24par and T24res PARP-1 (116 kDa) and cleaved PARP-1 (89kDa)
(b) Coomassie Brilliant Blue

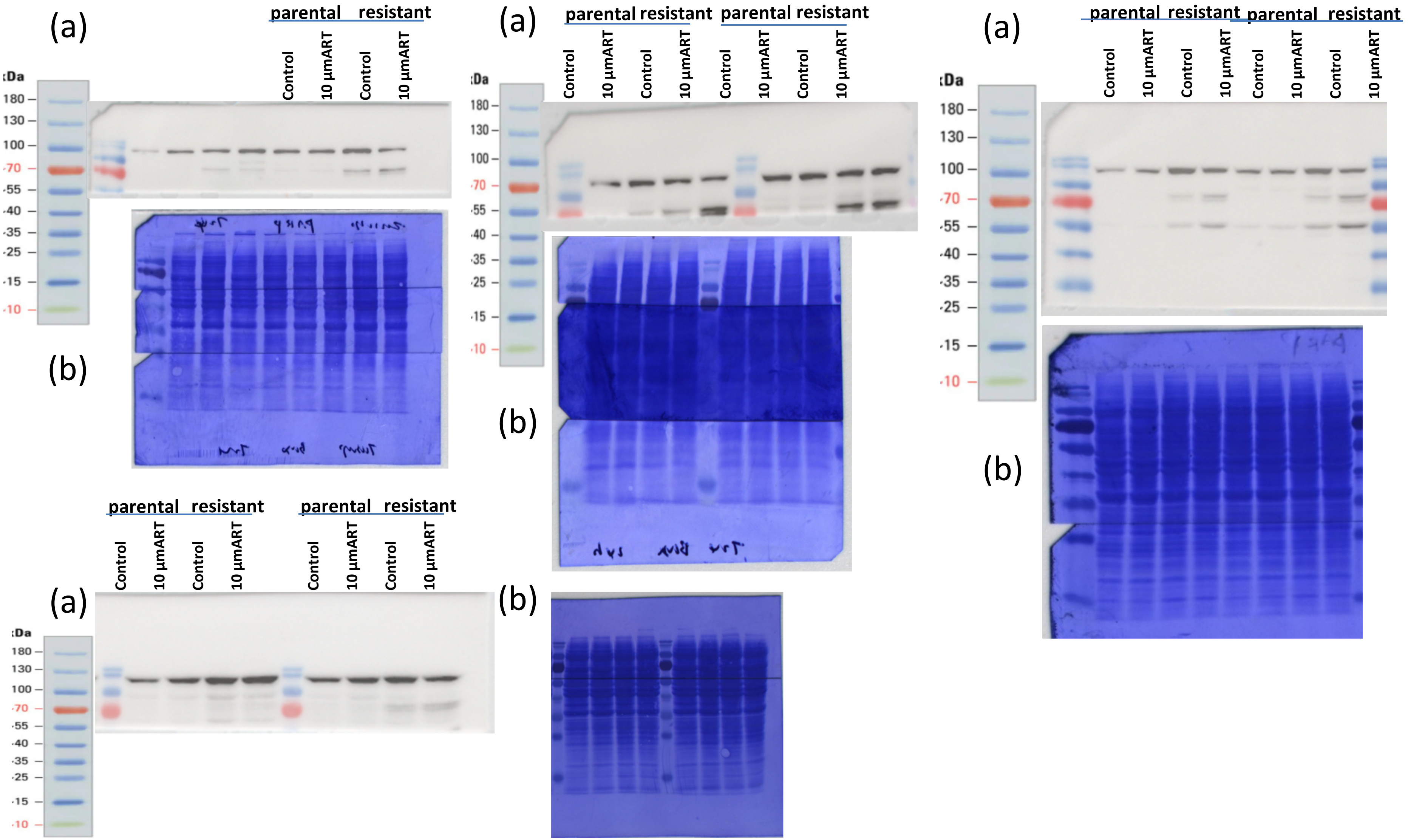

Figure S2.1: Detailed information about Figure 6 - Protein expression profile of DNA damage repair and apoptosis associated proteins in parental and resistant T24 cells. Protein expression of PARP-1 and cleaved PARP-1 (a), corresponding Coomassie blue staining of total protein (b).

Figure S2.2 (a) T24par and T24res, caspase 3 (35 kDa)
(b) Coomassie Brilliant Blue

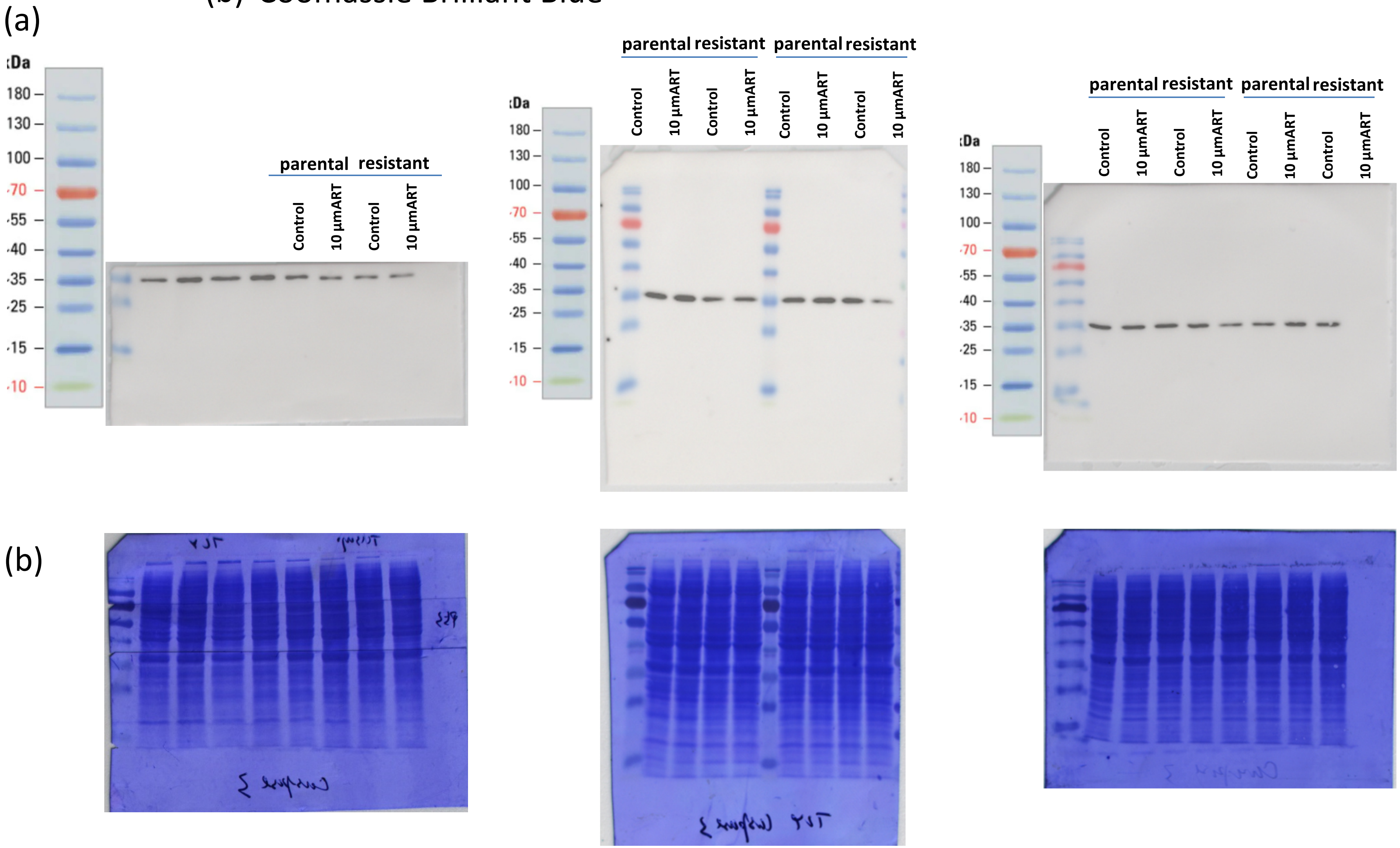

Figure S2.2: Detailed information about Figure 6 - Protein expression profile of DNA damage repair and apoptosis associated proteins in parental and resistant T24 cells. Protein expression of caspase 3 (a), corresponding Coomassie blue staining of total protein (b).

Figure S2.3 (a) T24par and T24res, caspase 8 (57 kDa)
(b) Coomassie Brilliant Blue

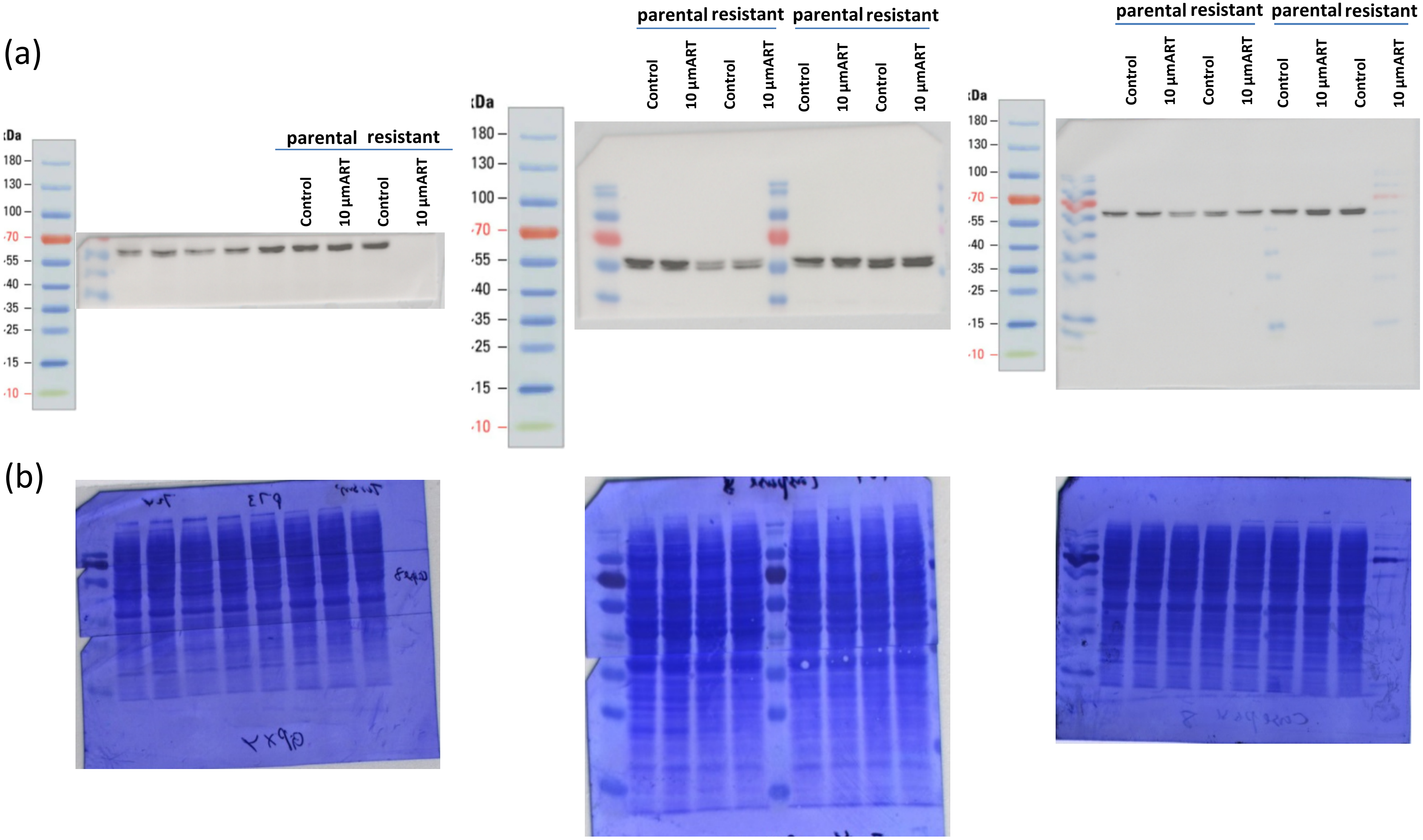

Figure S2.3: Detailed information about Figure 6 - Protein expression profile of DNA damage repair and apoptosis associated proteins in parental and resistant T24 cells. Protein expression of caspase 8 (a), corresponding Coomassie blue staining of total protein (b).

Figure S2.4 (a) T24par and T24res, Bcl-2 (28 kDa)
(b) Coomassie Brilliant Blue

(a)

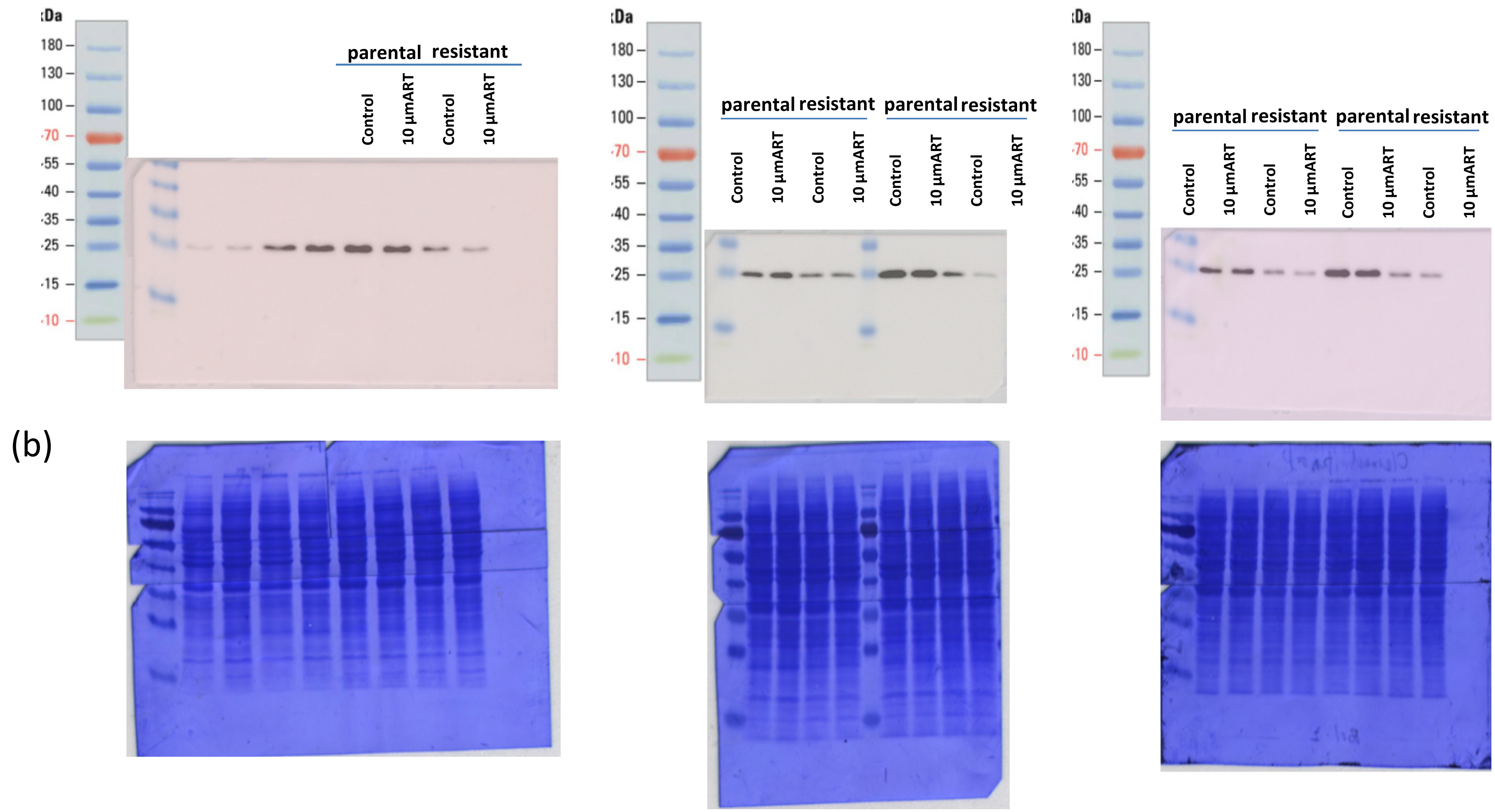

Figure S2.4: Detailed information about Figure 6 - Protein expression profile of DNA damage repair and apoptosis associated proteins in parental and resistant T24 cells. Protein expression of Bcl-2 (a), corresponding Coomassie blue staining of total protein (b).

Figure S2.5 (a) T24par and T24res, Bax (20.5 kDa)

(b) Coomassie Brilliant Blue

(a)

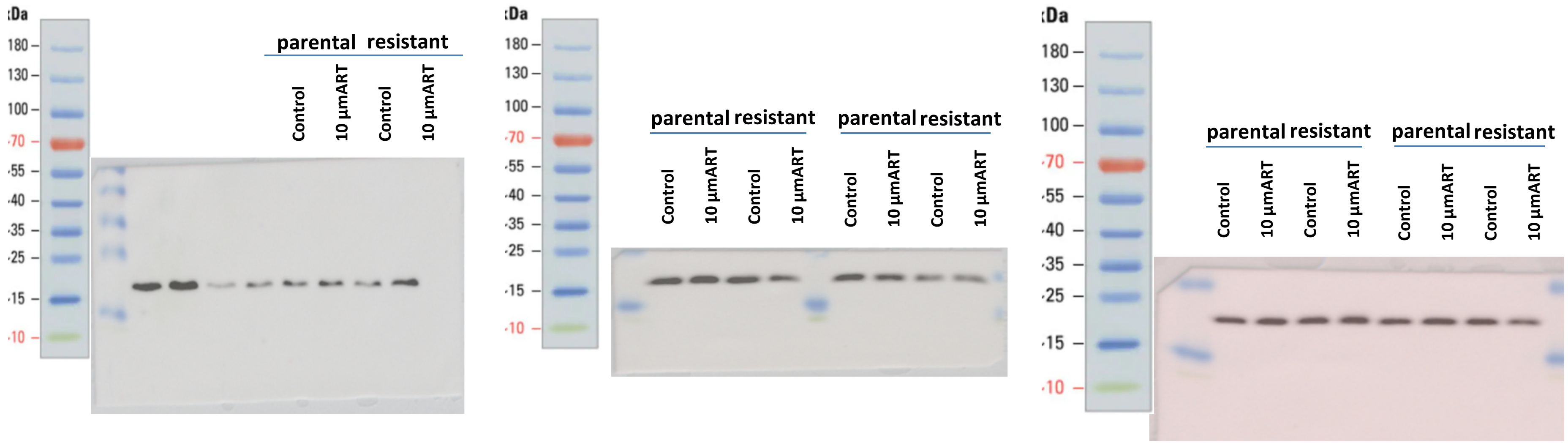

(b)

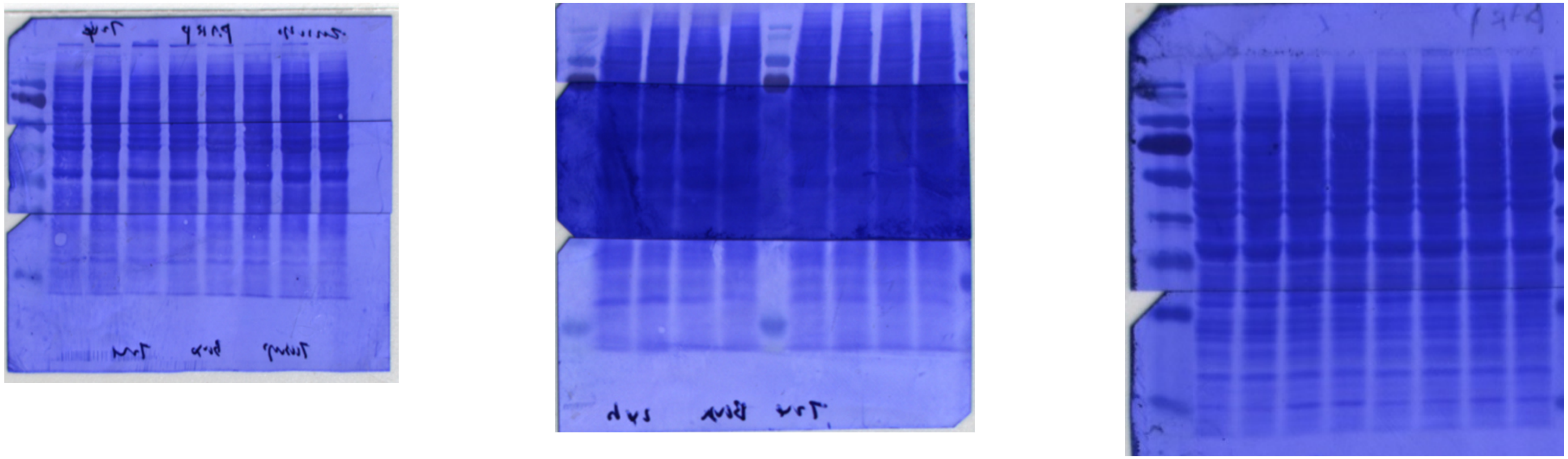

Figure S2.5: Detailed information about Figure 6 - Protein expression profile of DNA damage repair and apoptosis associated proteins in parental and resistant T24 cells. Protein expression of Bax (a), corresponding Coomassie blue staining of total protein (b).

Autophagy related proteins

Figure S3.1 (a) T24par and T24res, LC3B (17,19 kDa)
(b) Coomassie Brilliant Blue

(a)

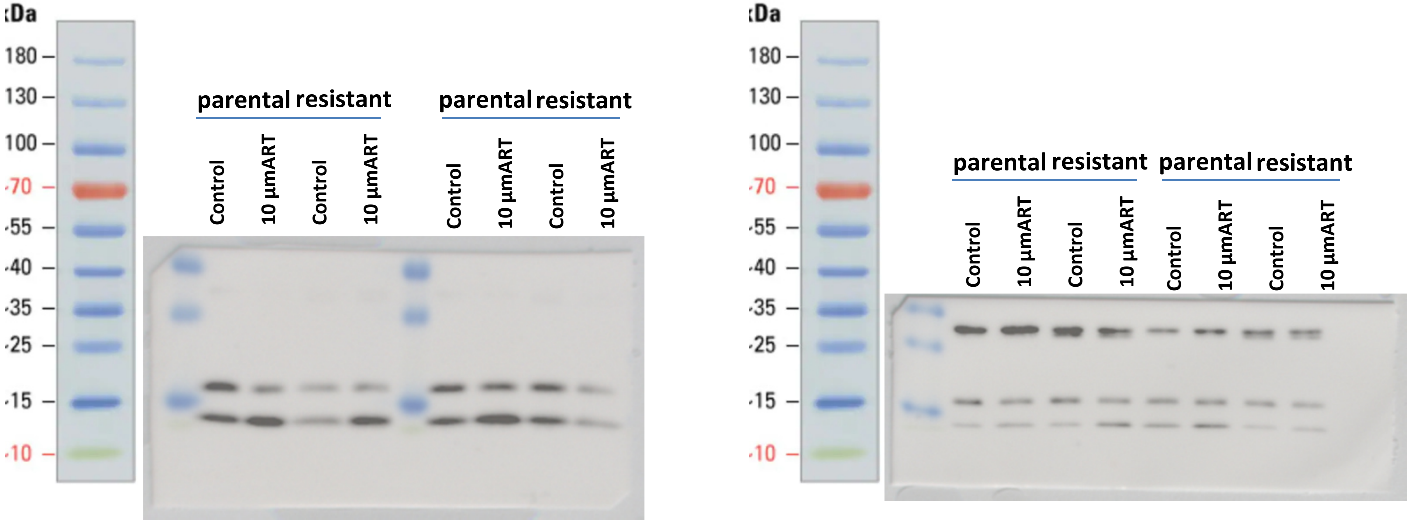

(b)

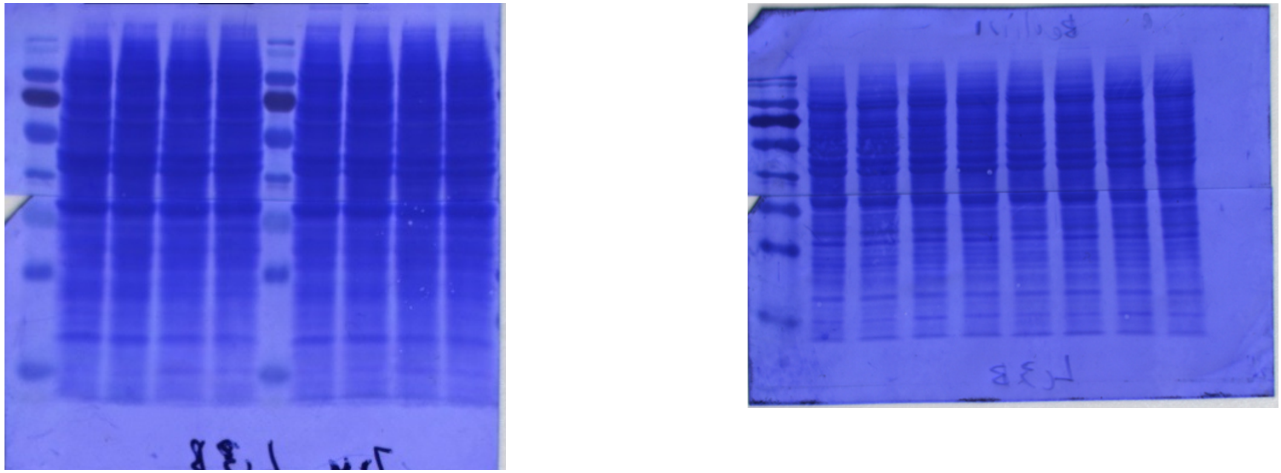

Figure S3.1: Detailed information about Figure 7 - Protein expression profile of autophagy associated proteins in parental and resistant T24 cells. Protein expression of LC3B (a), corresponding Coomassie blue staining of total protein (b).

Figure S3.2 (a) T24par and T24res + HCQ, LC3B (17,19 kDa)
(b) Coomassie Brilliant Blue

(a)

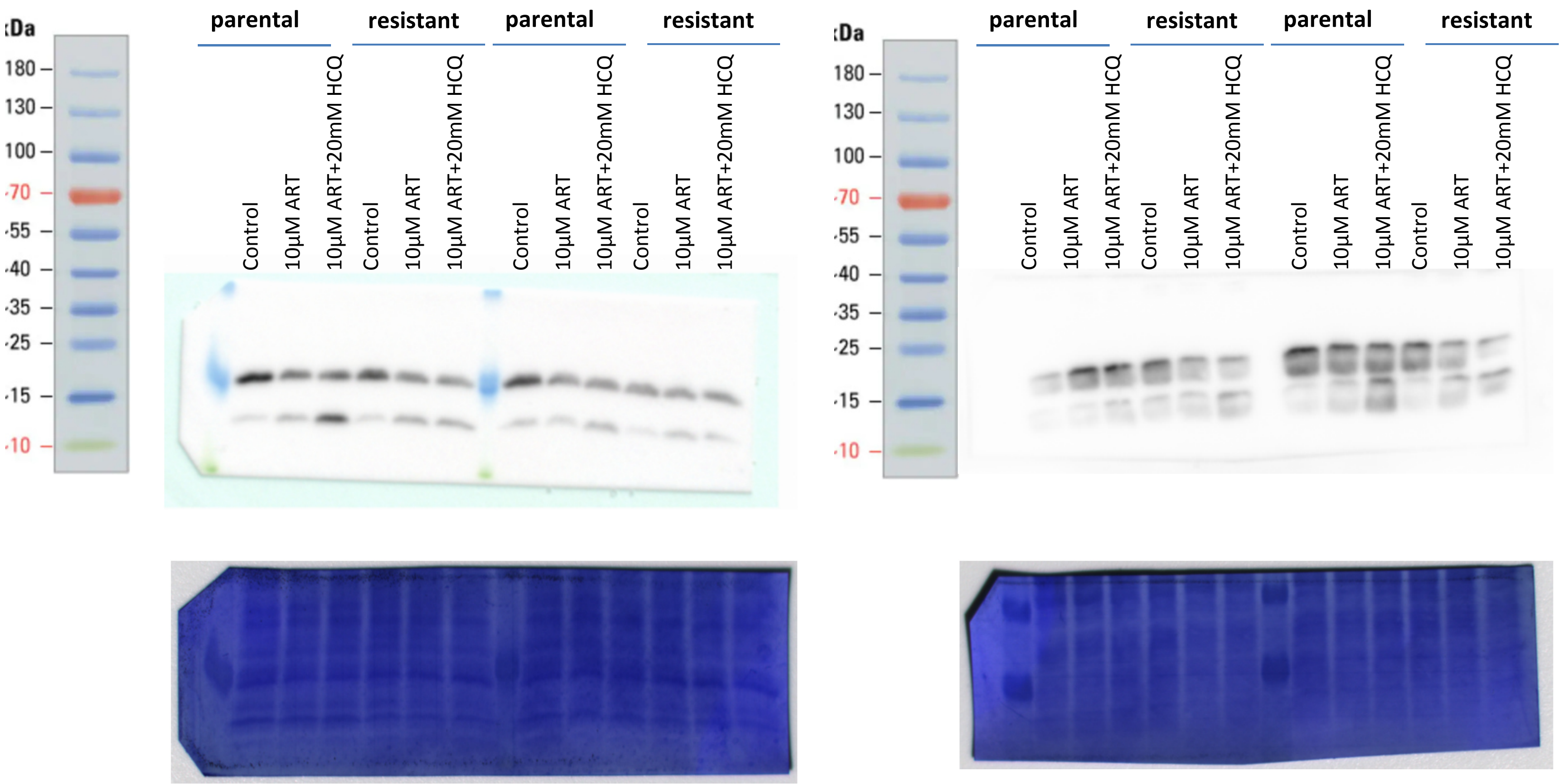

(b)

Figure S3.2: Detailed information about Figure 7 - Protein expression profile of autophagy associated proteins in parental and resistant T24 cells. Protein expression of LC3B (a), corresponding Coomassie blue staining of total protein (b).

Ferroptosis related protein

Figure S4 (a) T24par and T24res, GPX4 (24 kDa)
(b) Coomassie Brilliant Blue

(a)

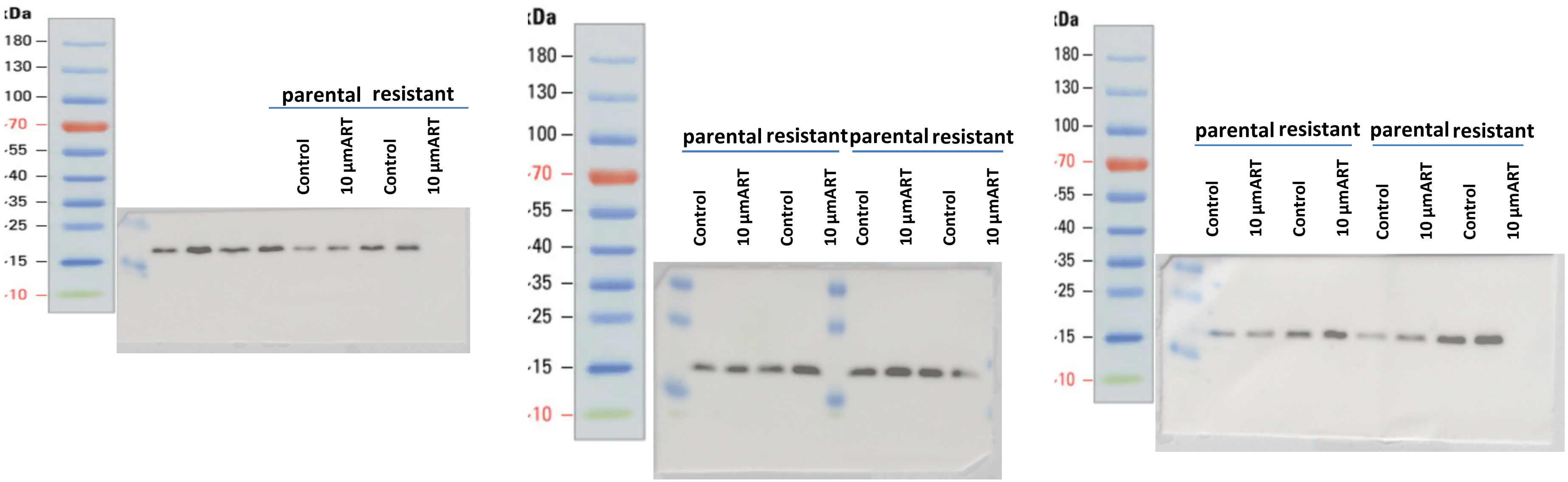

(b)

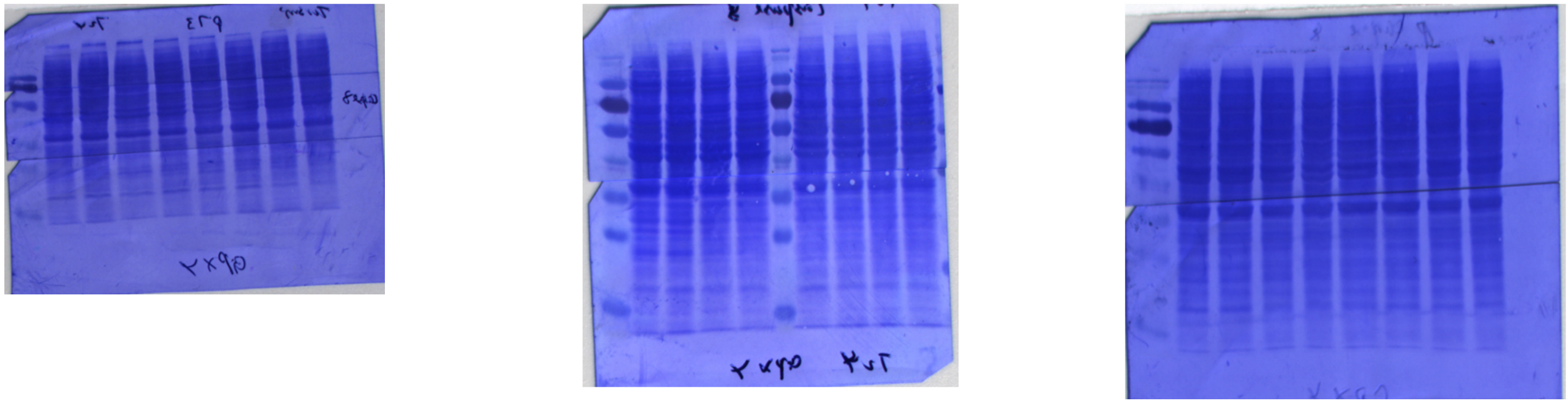

Figure S4: Detailed information about Figure 8 - Protein expression profile of ferroptosis associated protein in parental and resistant T24 cells. Protein expression of GPX4 (a), corresponding Coomassie blue staining of total protein (b).
